# From Verbal Reports to Personalized Activity Trackers: Understanding the Challenges of Ground Truth Data Collection with Older Adults in the Wild

**DOI:** 10.1145/3731749

**Published:** 2025

**Authors:** HOSSEIN KHAYAMI, LINING WANG, YOUNG-HO KIM, BONGSHIN LEE, DAVID E. CONROY, AMANDA LAZAR, EUN KYOUNG CHOE, HERNISA KACORRI

**Affiliations:** University of Maryland, College Park, USA; University of Maryland, College Park, USA; NAVER AI Lab, Republic of Korea; Yonsei University, Republic of Korea; University of Michigan, USA; University of Maryland, College Park, USA; University of Maryland, College Park, USA; University of Maryland, College Park, USA

**Keywords:** Older Adults, Seniors, Self Tracking, Human Activity Recognition, Machine Learning, Smartwatches

## Abstract

Tracking activities holds great potential to improve the well-being of older adults, yet the accuracy of activity trackers for this demographic remains in question. Evaluating this accuracy requires ground-truth data directly from older adults, which has largely been gathered in controlled laboratory settings or labeled by researchers. Moreover, considering the diversity in older adults’ activity engagement and tracking preferences, personalized activity tracking appears necessary. We demonstrate that older adults can benefit from personalized activity trackers by showing that cadence thresholds for stepping intensities vary within this group. However, collecting ground-truth data from older adults in real-world settings poses unique challenges. This paper examines two sources of ground-truth labels for the smartwatch Inertial Measurement Unit (IMU) data collected with older adults. Using verbal self-reports and a thigh-worn activity tracker, we assess their viability as ground-truth sources in natural settings. Additionally, we evaluate the costs and benefits of triangulating these sources as a ground-truth proxy. Our findings reveal two main costs: data shrinkage and notable effort from both contributors and data stewards. Simultaneously, we observe improved data quality and a greater ability to identify error sources when evaluating a trained model.

## 1 Introduction

Human activity tracking devices are becoming increasingly popular for monitoring health, enhancing fitness, and supporting independent living. Effectively training, benchmarking, and personalizing these human activity recognition (HAR) systems requires accurately labeled data that captures everyday movements in natural environments [36]. These environments often differ significantly from controlled settings, making it crucial to collect data that reflects real-world conditions. Yet, obtaining ground truth for activity recognition in such settings is challenging [50, 84, 90]. For instance, direct observation or video recording of daily activities by researchers, the data stewards, is neither ethical nor practical [9, 62, 65]. In addition to privacy and other ethical concerns, real-world human activity data rarely conforms to binary categories, and the notion of a singular “truth” is increasingly debated [12]. For example, whether reclining on a couch or lying with an upright upper body is classified as *sitting* or *lying* can vary by interpretation. This ambiguity highlights discrepancies between annotation sources and the inherent subjectivity in activity classification [12]. One potential approach is to rely on annotations reported by users, the data contributors, to address these discrepancies. However, to date, we lack an understanding of the challenges involved in extracting accurate and fine-grained activity labels and time spans from such high-level user reports. This issue is particularly pronounced when working with older adults, as we are still exploring their preferred and accessible methods of interaction for data labeling [53, 76, 93, 95].

In this work, we leverage data from a previous study [53] to explore challenges in obtaining ground-truth labels for this population in real-world settings. We triangulate two data sources: a thigh-worn sensor such as activPAL [3], considered state-of-the-art by kinesiologists [15, 40, 41], and verbal reports recorded via a smartwatch app in users’ natural environment.

Building on the idea that older adults’ self-reported activity labels can personalize activity trackers [53], we pose the following research question: **RQ1**: *Are cadence intensity thresholds for older adults variable to require personalization?* We focus on cadence since commercial activity trackers, typically trained on younger individuals, primarily differentiate walking from running. Prior research suggests that movement variability for the same activity is higher among older adults [19, 48, 52]. We examine challenges in obtaining ground-truths stepping intensity thresholds—low, moderate, and vigorous (often equated with running). Expanding beyond stepping, we investigate other common activities (lying, sitting, standing, and cycling) through additional research questions: **RQ2**: *What are the costs associated with obtaining a consensus between thigh-worn sensors and verbal reports as a proxy for ground-truth activity labels?* and **RQ3**: *What are the relative gains of consensus labeling with thigh-worn sensors and verbal reports for benchmarking an activity classifier?*

To address these research questions, we annotate and analyze real-world activity data from Kim *et al*. [53], collected over one week with 13 older adult participants. Participants wore both an activPAL thigh-worn sensor and a smartwatch. The activPAL, typically worn 24/7, provides built-in activity recognition including step count per minute and Metabolic Equivalent of Task (MET) values, which indicate activity intensity. From wake-up to bedtime, participants wore the smartwatch and reported activities via voice recordings using a smartwatch app. They could either initiate recordings or respond to Ecological Momentary Assessment (EMA) prompts, providing details such as activity type, timespan, and intensity. Kim *et al*. [53] categorized the verbal reports semantically and analyzed participants’ qualitative feedback. We extend this analysis with fine-grained, minute-level annotations essential for training and evaluating activity recognition models. Specifically, we annotate verbal reports at one-minute intervals and map them to data from the thigh-worn device, the smartwatch’s built-in sensors (accelerometer, gyroscope, and heart-rate), and activity predictions from the Google API.

We then provide evidence that cadence thresholds in older adults across low, moderate, and vigorous stepping are distinct and can benefit from personalization. Specifically, we observe that thresholds between Low to Moderate Stepping are in the wide range of 80–105 steps/min for the older adults in our study. Our observations indicate the potential for automatically estimating these personalized stepping boundaries by combining cadence with exertion information extracted from verbal reports or sensors that capture elevation, temperature, humidity, as well as heart and respiratory rate.

We find two main costs associated with obtaining ground-truth data using a consensus approach. The first relates to data shrinkage, with only one-third (34%) of the IMU data being labeled with consensus overall. This cost was evident among all participants and activities with contributing factors being both lack of labeled data from verbal reports and lack of agreement between the thigh-worn sensor and verbal reports. We find that this data shrinkage was more prevalent in low-intensity activities such as lying, sitting, standing, and low stepping.

The second cost relates to the level of effort required from both data contributors and data stewards. Beyond the inherent cost of privacy associated with the 24/7 wear of a sensor and time spent reporting, we provide evidence on additional costs for older adult data contributors associated with the adherence to wearing a sensor, the learning curve with the smartwatch, the cognitive load on recalling of activities and experiencing interruptions. Our results demonstrate that this cost of effort was divided, with the data stewards spending significantly more time (more than 300 hours) and cognitive load extracting the consensus data, with an outcome (321 hours and 35 minutes of consensus labeled data) that remains somewhat error-prone as they are called to make decisions and assumptions on both activity time spans and labels.

We identify two key gains from using consensus labeling by comparing the thigh-worn sensor, verbal reports, and consensus data to benchmark the performance of an activity tracker in the real world. First, model performance improves with consensus data, especially for high-intensity activities like running and cycling (improves the AUC score from 0.72 to 1.00 for running and 0.91 to 0.99 for cycling). Additionally, consensus labeling helps distinguish between errors caused by inaccurate labels and those from the model itself. For example, using the thigh-worn sensor as ground truth fails to capture atypical postures, such as “sitting on a kitchen stool,” accurately. Differences in sensor placement also lead to discrepancies between model predictions and labels, as seen in cycling and stillness detection. Furthermore, consensus labeling mitigates issues from errors in verbal reports’ activity time cues, which makes relying solely on verbal reports as ground truth problematic. We also identified model errors, such as the Google API mistaking motorized tool vibrations for being in a vehicle, or briefly missed detecting being in a vehicle when it stopped or idling.

This work builds on a study first presented in Kim *et al*. [53]. It contributes empirical results to better understand the challenges of obtaining ground-truth activity data with older adults, as well as the opportunities for using consensus approaches that triangulate sensor data with subjective verbal reports. Specifically, it provides (1) a comparative overview of labeling approaches in activity data collection over the past decade; (2) an approach for obtaining consensus data as a proxy for ground-truth activity labels with older adults in real-world settings; (3) empirical evidence supporting the need for personalized cadence thresholds for older adults; (4) an estimate of costs associated with obtaining consensus between thigh-worn sensors and verbal reports; and (5) a demonstration of the comparative gains of using a consensus approach for benchmarking a HAR application.

## 2 Related Work

Prior HAR work addressing challenges in obtaining ground-truth labels tends to rely on datasets with video recordings collected in controlled or simulated real-world environments. These studies primarily focus on active learning, where algorithms select video subsets for annotation by non-expert crowdworkers *e.g*. [64, 98], or semi-supervised learning, which uses limited annotated data to automatically label larger datasets by integrating sensor data *e.g*. [5, 47, 77, 84]. The closest work to ours is Hu *et al*. [46], which collected data in near real-world setting without video recordings, approximating ground-truth labels by combining multiple sensor sources. However, unlike our approach, their work excludes self-reported data and focuses on semi-supervised learning with small, fine-grained datasets labeled by data stewards, alongside larger coarse-grained data. Beyond ground-truth challenges in HAR, we review research on understanding older adults’ activities and developing recognition models. We position our contributions within this literature by examining studies that collect data from older adults, with a particular focus on their annotation and labeling methods.

### 2.1 Understanding Older Adults’ Activities

#### The Importance of Understanding Older Adults’ Activities.

Understanding older adults’ activities is vital for promoting healthy aging and improving quality of life [17, 37, 82]. The benefits fall into three areas: (a) supporting well-being by encouraging meaningful activities, (b) providing healthcare professionals with insights for effective interventions, and (c) guiding the design of age-friendly technologies. Regarding (a), community-based interventions that encourage active participation in recreational activities [7], incorporating physical activities into online social experiences [16], promoting engagement in meaningful activities [6, 74] have proven beneficial for older adults’ health. Concerning (b), healthcare professionals benefit from research into the impact of disabilities, dementia, depression [73], physical activity, and sleep on well-being [38, 82]. Finally, (c) several studies highlight human-computer interaction challenges in developing age-friendly technologies [37, 76, 93].

#### Type of Activities that are Common / More Interesting for Older Adults.

A great body of research is trying to extract activities that are more common among older adults [11, 25, 32, 43, 44, 51, 53, 58, 67, 75, 81, 87]. Lazar and Nguyen [58] found a variety of leisure activities, including watching TV, reading, gardening, and pet care. Additionally, Cho et al. [25] analyzed passive and active leisure activity frequencies. Through an experience sampling method using a smartwatch, Kim et al. [53] identified 29 distinct activity types, organizing them into nine high-level categories, with housekeeping-related activities reported as the most frequent. Wang et al. [93] identified several types of activities that older adults find meaningful, including those related to physical health, mental well-being, cognitive health, social connectedness, and basic needs. Additionally, the authors highlighted the importance of low-exertion activities, such as indoor ambulation, stretching, and household chores, which are often overlooked but contribute significantly to older adults’ physical health.

#### Feasibility and Compliance of Using Mobile and Smartwatches to Record Older Adults’ Activities.

Studies have explored the feasibility of collecting activity data using diverse methods [5, 95]. Although some efforts such as IMUTube have utilized virtual IMU data from online videos to train machine learning models [56, 57], ground-truth data remains essential for evaluating these models. Few studies focus on in-situ data labeling with older adults. Chung et al.[28] found that while older adults valued smartwatches for tracking health, privacy concerns and readability posed challenges. Maher et al.[63] demonstrated that ecological momentary assessment (EMA) effectively captured older adults’ physical activity patterns, informing future interventions to promote physical activities. Cristescu and Bajenaru [29] developed a smartwatch adoption model considering design and usability factors to enhance wearables’ appeal. Klocek et al.[55] evaluated mobile devices’ feasibility for real-time monitoring in Czech older adults. Voice-based journaling, which is believed to lower the technology barrier [76], has been used to collect activity and posture data [53, 59] and has been examined to understand older adults’ meaningful activities [93].

### 2.2 Labeling Older Adults’ Activities

In this section, we review prior HAR work with older adults to capture the breadth of these data annotation approaches and contextualize them with information about the data contributors, activities, devices, and sensors employed. Table 1 provides a comparison of studies over the last decade and contrast to our work.

#### Labeling and Ground Truth.

At a high-level, we observe two common approaches. The first includes annotations provided by the data contributors (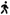), typically study participants. In few cases, older adult data contributors either label their activities from a predefined list (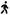 *Pick Activity*) *e.g*. using a mobile device [30, 72, 89] or write them down 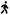 *Note Down*) [45]. The more common approach with this population involves annotations from data stewards (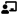), typically the researchers collecting the data. Data stewards label activities by observing the data contributors either in real-time (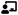 *Label in Sync*) or through recorded videos (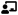 *Label Videos*). As mentioned above, only in Hu *et al*. [46], we find that data stewards attempt to obtain a consensus (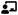 *Triangulate*) by combining video labels with sensors (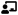 *Use Sensors*).

**Fig. 1.**
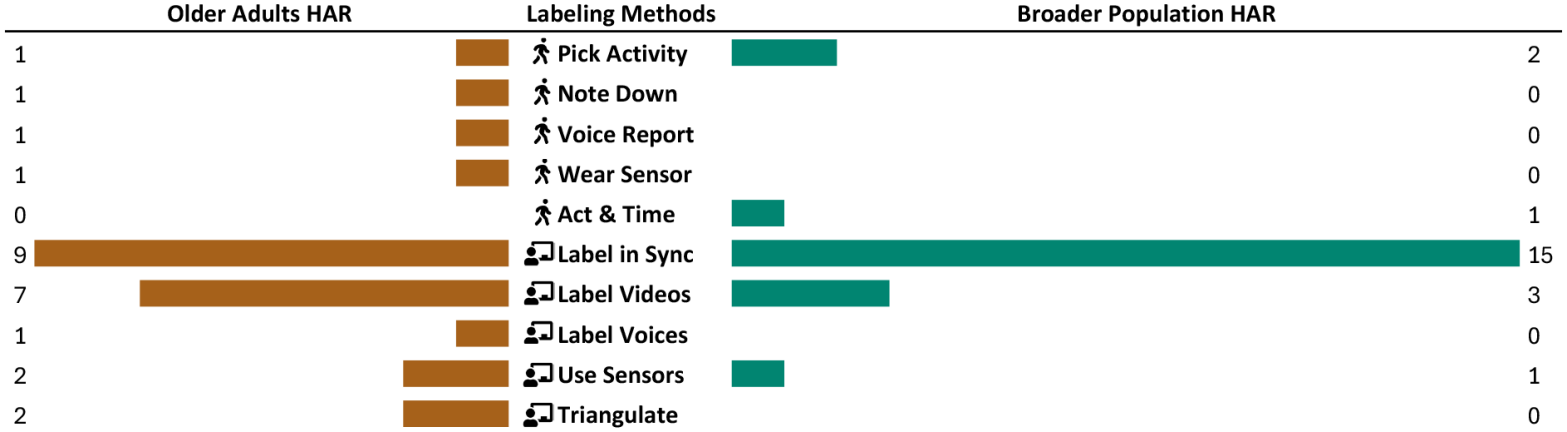
The distribution of labeling methods utilized across studies focusing on human activity recognition (HAR), comparing data from older adults with the broader population. In most studies, data stewards (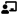) label the activities either in sync or by watching recorded videos. Only a few managed to have data contributors (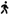) do the labeling themselves.

When comparing these labeling strategies to a representative sample from HAR literature covering a broader population (not just older adults) during the same period (see Figure 1), we observe similar trends. However, in older adults HAR studies, a greater proportion of data stewards choose to label videos. Notably, one strategy seen in broader HAR–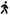 *Act & Time*, where data contributors are asked to both act on a predefined order and time their transitions *e.g*., [14]– is absent in older adult HAR. To eliminate video recordings while collecting real-world data, in our study we share the labeling burden with older adult contributors by triangulating voice recordings of activities (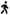 *Voice Reports*, 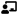 *Label Voices*) and wearable sensor data (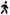 *Wear Sensor*, 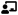 *Use Sensors*).

#### Data Contributors.

Similar to our work, HAR data collections with older adults typically include participants aged 60 years and older. Few (*e.g*., Schrader et al. [80] and Mardini et al. [66]), recruited a more diverse cohort comprising both younger and older adults. Typically, older adult data contributors are identified as women and men with no mention of non-binary individuals. However, the size of data contributors varies across studies. While the size of our study aligns with most reported data collections (5–22), a few examples (*e.g*., [13, 36, 66]) involve hundreds of older adult data contributors. Surprisingly, in these instances, ground truth labels are obtained through real-time observations made by the data stewards.

#### Activities: Postures, Places, and Goals.

HAR data stewards typically focus on a predefined set of 5–15 activities for older adults, with most studies, including ours, labeling activities at the posture level such as lying, sitting, standing, and walking. Some studies targeted fewer but health-specific activities, such as falls [45], bed and chair exits [79], or functional dependence in shopping [36]. Studies with larger activity sets (*e.g*. 33) prioritized physical and metabolic performance estimation [13, 49], limiting recognition to broad categories such as sedentary vs. non-sedentary. In these studies, participants’ activities are typically limited to their home, community spaces, or controlled settings (labs, clinics, hospitals), where data stewards observe and label activities in real-time or via video review. This remains the case even when data contributors provided labels ([45, 72]). However, older adults’ routines extend beyond these spaces and may include outdoor activities such as jogging, biking, and driving—activities we aimed to capture in real-world settings. Observations, whether direct or via video, are typically not feasible in these environments. However, one study had data stewards follow and observe older adults’ shopping activities [36]. In contrast, our work focuses on unrestricted real-world activities in indoor and outdoor settings, relying on older adults’ input for data labeling.

#### Data: Sensors and Devices.

Data stewards used various devices to track activities, including wearables, mobile phones, and ambient sensors. Wearable devices equipped with Inertial Motion Unit (IMU) sensors—such as accelerometers, gyroscopes, and magnetometers—capture body joint motions at the attachment points. Many wearables also include sensors for heart rate, air pressure, and skin temperature, as seen in smartwatches and fitness bands. Some studies employ more advanced measurements such as metabolic equivalents (MET), requiring bulkier equipment that limit accessibility. Indeed, certain devices used in [34, 79, 80] required specialized mounting or multiple body attachments, potentially affecting participants’ natural behavior. Ambient sensors can provide location data but require additional setup to integrate with wearables for activity recognition and are restricted to indoor use. To minimize behavioral impact, our study opted for sensors available in smartwatches and wearables such as activPAL, which offer convenient placement.

## 3 Methods

This work is part of a larger project aimed at understanding older adults’ activities. In this article, we address the challenges of obtaining ground truth labels in real-world settings by using verbal reports, sensor predictions, and our method of combining both. We operate under the assumption that activity trackers should consider the variation in movement patterns among older adults, and we investigate this assumption. To this end, we asked our participants to wear a smartwatch on their wrist and a physical activity tracker sensor called activPAL [3] on their thighs for a week. They logged their daily activities verbally using our smartwatch application, which also records IMU sensor data.

**Fig. 2.**
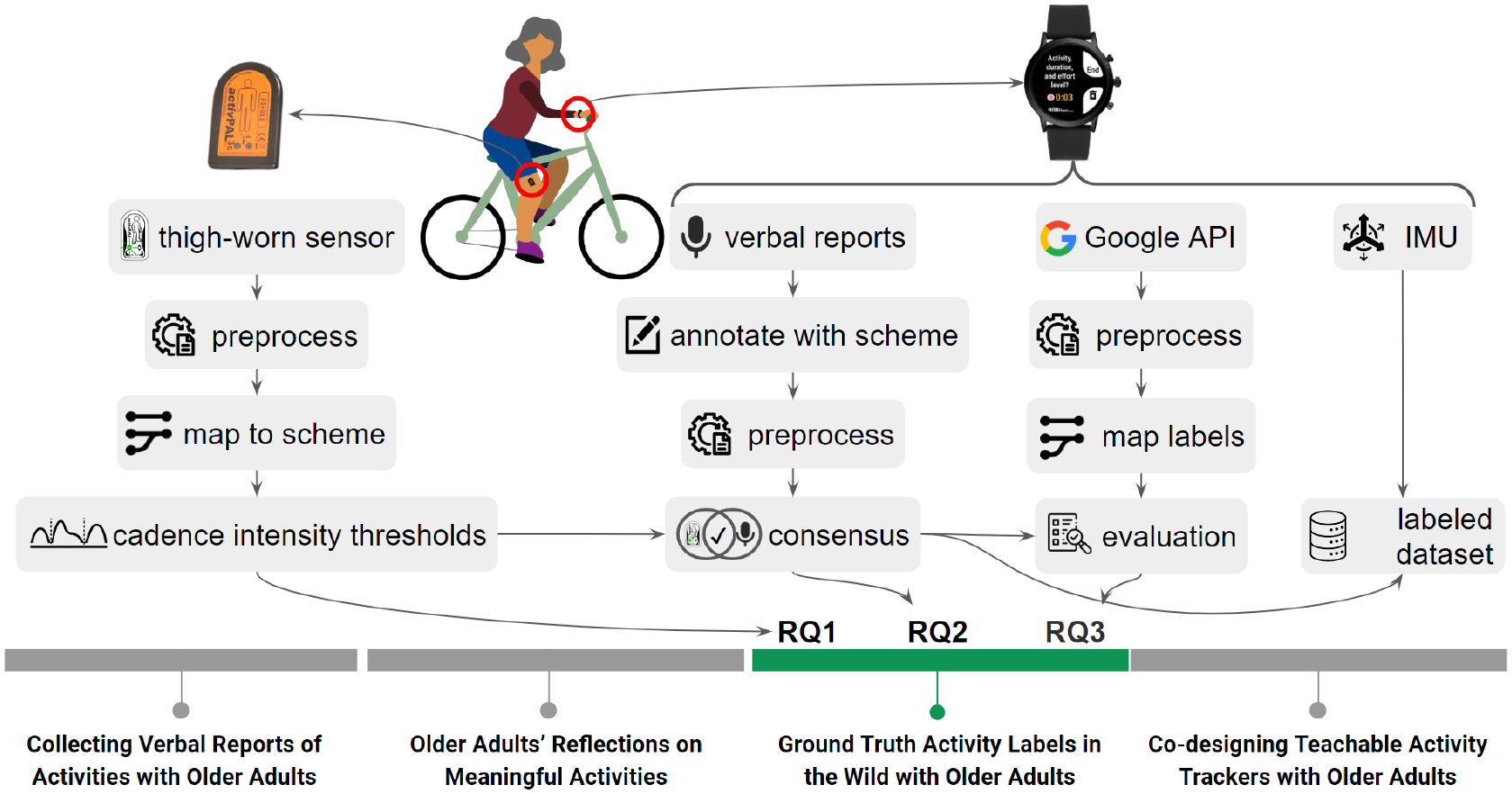
Sensor placement and system overview. The upper section illustrates sensor placements and data sources, while the middle section outlines analysis steps addressing our research questions. The green bar in the lower section highlights this paper’s scope and its connection to prior work by Kim *et al*. [53] and other ongoing efforts within the larger project.

We use the collected verbal reports and the thigh-worn sensor data to explore the challenges of real-world ground-truth activity data collection activity with older adults (see Figure 2 for an overview). Specifically, we annotate the verbal reports to extract activity labels and propose a personalized cadence threshold method for stepping intensities. We then investigate whether these thresholds are so variable that they need to be personalized (RQ1). We mapped activPAL and verbal reports to a shared representation, combining them to find a consensus and analyze labeling costs and challenges (RQ2). Finally, we investigate the possibility of using our ground truth labels to evaluate an activity tracker application and observe the relative gains (RQ3). This study was approved by the Institutional Review Board of the University of Maryland, College Park (IRB#1132164–15).

In Section 3.1, we explain our user study, including participants’ information, study duration and environment, and data collection. Section 3.2 explains how we preprocess and annotate reports from our data sources to our shared representation activity labels. Section 3.3 describes our data analysis to answer our research questions. Specifically, we analyze the achieved personalized cadence thresholds, and the costs and relative gains involved in the consensus labeling method to reach ground truth labels.

### 3.1 Data Collection

#### 3.1.1 Participants.

A total of 13 older adult participants were recruited. All were selected based on the following criteria: (1) willingness and comfort in verbally reporting daily activities in English; (2) lack of severe speech, cognitive, or motor impairments; and (3) right-handedness. Participants’ ages ranged from 61 to 90, with an average of 71.08. Table 2 presents their demographics. Among the 13 participants, 3 are men and 10 are women, with none identifying as non-binary. Participants had varying familiarity with machine learning. About one-third were unfamiliar with machine learning, another third had heard of it but did not understand its functionality, and the rest had a general understanding of its principles. For instance, P4 mentioned, *“They built Watson to beat Kasparov in chess,”* while P6 suggested more detaile, descriptive input could enhance AI learning capabilities.

#### 3.1.2 Apparatus.

Building on prior efforts to minimize data collection burden on older adults [8, 18, 26, 27, 31, 83, 95], Kim *et al*. [53] designed a smartwatch application on the Fossil Gen 5 Android smartwatch, chosen for its large screen and physical buttons. The app enables voice input via a tap or button press.

#### 3.1.3 Procedure.

Data collection occurred over one week during participants’ waking hours in their natural environments. It continued uninterrupted as participants moved through their usual living spaces or went out.

##### Onboarding.

Each participant attended a 1:1 introductory session where they were trained to use the smartwatch and attach the activPAL sensor. The researcher explained the study purpose and guided them through setting up the smartwatch, attaching the activPAL to their thigh, and ensuring device connectivity (see Figure 2 for sensor placements). The study included a 4-day adaptation period for participants to become comfortable using and charging the devices. They were instructed on recording verbal reports via the smartwatch, providing a one-to-two sentence description of their activities using examples as guidance.

##### Verbal Activity Reports.

Participants reported activities by voice, specifying the activity, duration, and effort level. Reports could be submitted anytime or in response to hourly prompts during wake time, if no recent entry had been logged and the watch was worn. Transcribed entries with corresponding timestamps are stored.

##### Thigh-Worn Sensor.

The activPAL thigh-worn activity tracker served as a second labeling source. Data were retrieved by connecting the sensor to a computer extracting 1-minute epochs to align with other data sources.

##### Smartwatch Sensor Data.

The smartwatch app collects IMU data—acceleration, rotation, and gyroscope—at 50 Hz for 10 seconds every minute (500 samples/min) and logs heart rate and step count in one-minute bins.

##### Google API Labels.

The smartwatch, equipped with the Google Activity Recognition API, records one-minute activity labels. Data, including API labels and IMU readings, were excluded when the watch was not worn.

### 3.2 Data Preprocessing and Annotation

**Fig. 3.**
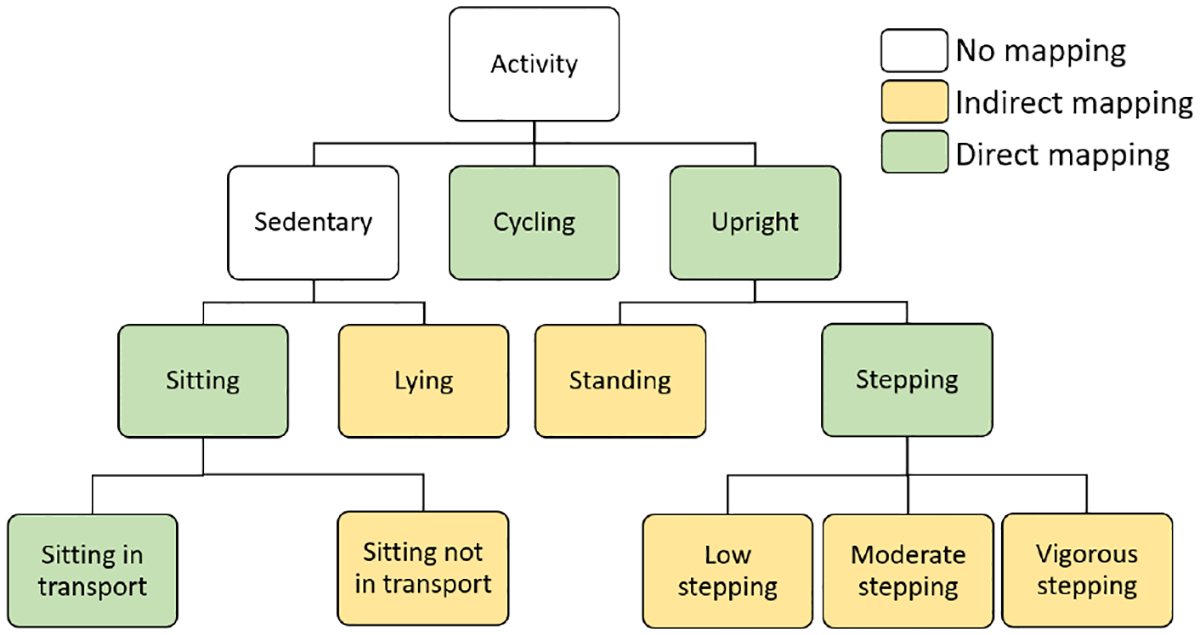
Mapping thigh-worn sensor data into the hierarchical activity scheme. Most labels map directly (Sitting, Sitting in transport, Cycling, Upright, and Stepping), while others (Lying, Sitting not in transport, and Standing) are inferred by combining labels. Fine-grained stepping labels are determined through personalized cadence analysis.

We preprocess thigh-worn sensor and annotate verbal reports to achieve a shared representation of activity labels, which we map into a hierarchical activity scheme, shown in Figure 3. Our scheme is informed by prior work [39], where activities are categorized based on body posture such as sedentary, upright, and cycling.

#### 3.2.1 Thigh-worn Sensor Reports.

Upon loading the sensor data, we use the activPAL software [3] to generate reports at 1-minute epoch, matching the Google API labels’ granularity. As shown in Figure 3, many of activPAL labels directly map to our hierarchical activity scheme, includingSitting, Sitting in transport, Cycling, Upright, and Stepping. However, activPAL reports allow a minute-long epoch to contain multiple labels, the duration of each indicated in seconds. For a given epoch, we assign the label with a duration exceeding 30 seconds. A small number of epochs (see Results) that don’t meet this criterion are excluded.

ActivPAL labels that cannot be directly mapped into our hierarchical activity scheme are inferred as follows:

**Lying**: activPAL provides fine-grained labels for lying indicated as primary and secondary. We aggregate them into a single label. If their combined duration within a minute exceeds 30 seconds, we assign it to the epoch.
**Sitting not in transport**: activPAL lacks labels for sitting outside transport but uses ‘sedentary’ as an umbrella term for this posture, including seated transport. We infer sitting duration outside transport within an epoch by subtracting seated transport time from total sedentary time. If the remaining duration in a minute exceeds 30 seconds, we assign this label to the epoch.
**Standing**: activPAL lacks direct labels for standing but uses ‘upright’ as an umbrella term for other activities with this posture. We infer standing duration within an epoch by subtracting cycling and stepping time from the total upright time. If the remaining duration in a minute exceeds 30 seconds, we assign this label to the epoch.
**Low Stepping, Moderate Stepping & Vigorous Stepping**: activPAL lacks direct labels to differentiate stepping intensity levels. However, this distinction–commonly seen in commercial products as walking versus running– is part of our hierarchical activity scheme. We used individual cadence distributions and verbal reports to determine cadence thresholds for Low, Moderate, and Vigorous Steppingas detailed in Section 3.3.1.

#### 3.2.2 Verbal Reports.

Transcribed participants’ reports contain two or more sequential activity events. In this paper, we define an *“activity event”* as a participant-defined activity mapped to a single label (*i.e*. singleton), multiple labels (*i.e*. composite), or none (*i.e*. undefined). When reports include transition time cues, we break them into individual rows and manually code the timing cues for start and end times. Participants were free to incorporate time cues creatively, making annotation highly subjective–akin to *“detective work”*. Patterns in time reporting style are presented in the Results. We exclude a small percentage of activity events (see Results) that are either undefined or lack inferable start and end times.

For instance, P4’s report, *“Biking for about 15 minutes, moderate intensity.”*, is coded as a singleton activity event with Cycling as the sole label, with the start time inferred from the recording time and duration. Another report from P13, *“I went for a brisk walk from 4:38 PM until 5:11 PM.”*, is mapped to Moderate Stepping with clear start and end times. Composite activity events include cases like *“playing golf”* (P1), mapped to Standing and Low Steppingin our hierarchical activity scheme. They also include multiple events without transition cues, such as *“For about the last 45 minutes …, I have been doing light housework, putting things away, and some yard work, mainly watering the sunflowers.”* (P11), which is also mapped to Standing and Low Stepping. Due to the limited postures in our hierarchical activity scheme, some events, like *“I’m going to swim laps for 15 to 20 minutes.”* (P5), are coded as undefined. Undefined events also arise when mapping becomes *“guess work”* rather than *“detective work”*, such as P6’s *“Just completed an hour of occupational therapy…”*, where suitable labels are unclear.

Given the subjectivity of this annotation process, three researchers (also authors) conducted an iterative process. One initially coded data for P1 and P2, then discussed the coding scheme with a second. A third researcher re-annotated P1 and P2 using finer-grained scheme. All three met to resolve challenging cases. For instance, we labeled *“walking a dog”* as a composite event of both Low and Moderate Stepping, *“stretching”* as undefined when posture was unspecified, and *“using a computer”* as Sitting not in transport unless a different posture was explicitly reported. The third researcher refined the scheme and applied it to the remaining data.

### 3.3 Data Analysis

We determine cadence thresholds for stepping intensities per participant and examine their variability (RQ1). Next, we derive consensus labels by triangulating labels from the thigh-worn sensor and verbal reports. We then analyze the consensus labeling process to assess the costs and relative gains of triangulation (RQ2 & RQ3).

#### 3.3.1 Stepping Thresholds:

Step count is commonly used to estimate metabolic equivalents (METs), with predefined thresholds classifying activity intensities as light (≤ 2.99 METs), moderate (3.00–5.99 METs), and vigorous (6.00–8.99 METs) [4]. This classification can help older adults track their progress toward health goals. The CDC recommends that adults 65+ engage in 150 minutes of moderate or 75 minutes of vigorous activity per week [22]. However, low-exertion activities, characterized by lower step counts, are more common among older adults [93]. Activity trackers, such as Fitbit, often undercount steps at slower walking speeds typical of this group [94]. Additionally, step counts for older adults may differ significantly from younger adults when running [86]. Even group thresholds may not accurately capture older adults’ activity intensities, as they exhibit more behavioral variability within their age group due to diverse aging trajectories [21, 85]. In this work, we investigate whether cadence intensity thresholds among older adults are so variable that they need to be personalized (RQ1).

To address this question, we manually determine stepping thresholds for each participant and compare them to activPAL’s built-in MET estimation and non-linear regression approaches based on step counts [4]. A kinesiology and human development researcher on our team manually reviewed each participant’s walking cadence distribution alongside their verbal reports. Specifically, the researcher looked for three distinct peaks in the cadence distribution for all stepping epochs (see Figure 6), indicating distinct low, moderate, and vigorous stepping cadences. Participants’ verbal reports were analyzed in two ways. First, we generated a word cloud from each participant’s verbal reports associated with labels of stepping. The word cloud provided a high-level snapshot of events per participant, where stepping occurred. It merely gave the kinesiologist a glimpse of how active a participant was. For example, P13’s word cloud highlighted instances of *“exercising”*, *“running”*, *“swimming”*, and *“riding”*. The word cloud was then enriched with a list of instances in which the participant described exertion, each linked to step count ranges, such as *“Walked dog for approximately 20 minutes, medium brisk pace.”* (P6) with a [82,112] step count range and *“I ran and did a … combination run walk. It was like kind of a moderate level of exercise …”* (P13) with a [112,164] step count range. The kinesiologist leveraged this list of (exertion instance, step count range) pairs to confirm that the thresholds from the peaks align with participants’ reports.

#### 3.3.2 Consensus Labeling: Costs of Aligning Thigh-worn Sensor and Verbal Reports as Ground Truth.

To capture ground truth data without video recordings, we use two complementary sources: the activPAL thigh-worn sensor [3] and participants’ verbal reports. The activPAL sensor, known for posture recognition [15, 40, 41], provides granular minute-level labels. Verbal reports, while covering longer periods (averaging 27 minutes per event), reflect participants’ personal semantics. Achieving a consensus between the two as a proxy for ground truth comes with costs. Through data analysis, we explore and, when possible, quantify these costs (RQ2).

Our analysis focuses on data shrinkage, examining its distribution across participants and activities to identify insightful patterns. To assess its impact on each activity, we compare thigh-worn sensor labels and verbal reports, generating a confusion matrix to show their alignment (see Section 4.2.1).

We examine the effort costs of capturing and annotating verbal reports, using the thigh-worn sensor, and triangulating both for consensus. Since annotating verbal reports is the most labor-intensive task for data stewards, we detail this process, which involves manual coding and partial automation. We first summarize participants’ time-reporting styles in a table, followed by statistics on singleton, composite, and undefined activity events. These quantify labeling challenges, highlighting cases requiring careful interpretation and educated guessing. Through visualization in Section 4.2.2, we illustrate the efforts involved for both data contributors and stewards.

#### 3.3.3 Google API Case Study: Gains of Consensus Labeling.

We evaluate the potential of using our consensus labeling as ground truth to analyze the performance of an activity tracker (RQ3)—specifically, the Google Activity Recognition API [1] on the smartwatch.

**Fig. 4.**
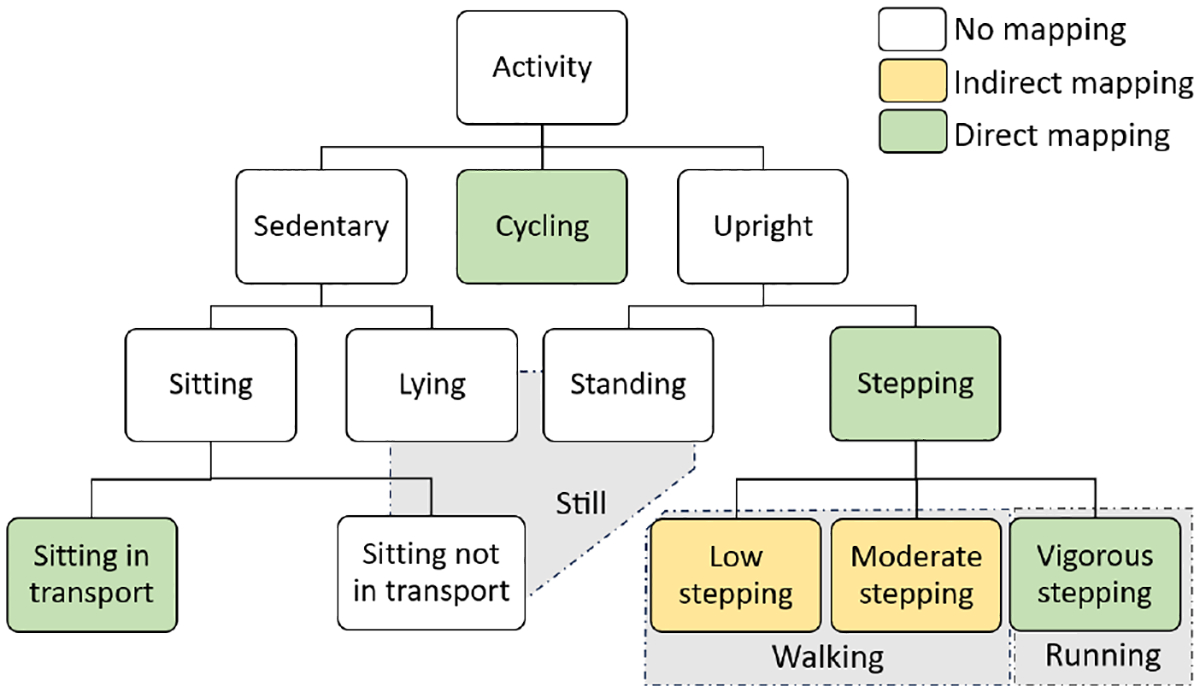
Mapping Google API data to the hierarchical activity scheme. Some labels, such as On_Bicycle (=Cycling), On_Foot (=Stepping), Running, and In_Vehicle (=Sitting in transport), map directly. When one-to-one mapping isn’t possible, we assign multiple hierarchical activity labels to broader Google API labels such as Still and Walking.

The Google API outputs confidence levels (0–100) for activities such as Still, In_Vehicle, On_Foot, Walking, Running, On_Bicycle, Tilting, and Unknown. Since Google API classes don’t directly map to our hierarchical activity scheme, we align labels as illustrated in Figure 4. We first plot Receiver Operating Characteristic (ROC) curves to determine optimal thresholds that maximize Youden’s J statistic (= *TPR* − *FPR*). For activities with a confidence level surpassing these optimal thresholds, we choose the corresponding Google API activities.

We evaluate the performance of the Google API activity recognition using the Area Under the Curve (AUC score), considering our consensus labeling as the ground truth. We compare its performance in a table against evaluations using only verbal reports or the thigh-worn sensor to see the effect of the labeling method on the perceived model performance. Since each labeling source has errors, we expect consensus labels to provide higher-quality data. By analyzing misalignments, we identify error sources in each method, demonstrating how consensus labeling offers deeper insights into errors.

## 4 Results

Over 7 days, we collected 2140 hours and 56 minutes (23 hours and 31 minutes per day per participant on average) of thigh-worn sensor data, capturing activities day and night as participants rarely removed the adhesive sensor patch. Smartwatch data totaled less than half (941 hours and 9 minutes, about 10 hours and 20 minutes per day per participant on average) since it was typically worn during the daytime. Participants’ verbal reports cover even less (522 hours and 3 minutes, 5 hours and 44 minutes per day per participant on average). They were collected via a total of 1241 voice entries in our smartwatch application.

### Excluded thigh-worn sensor data.

**Fig. 5.**
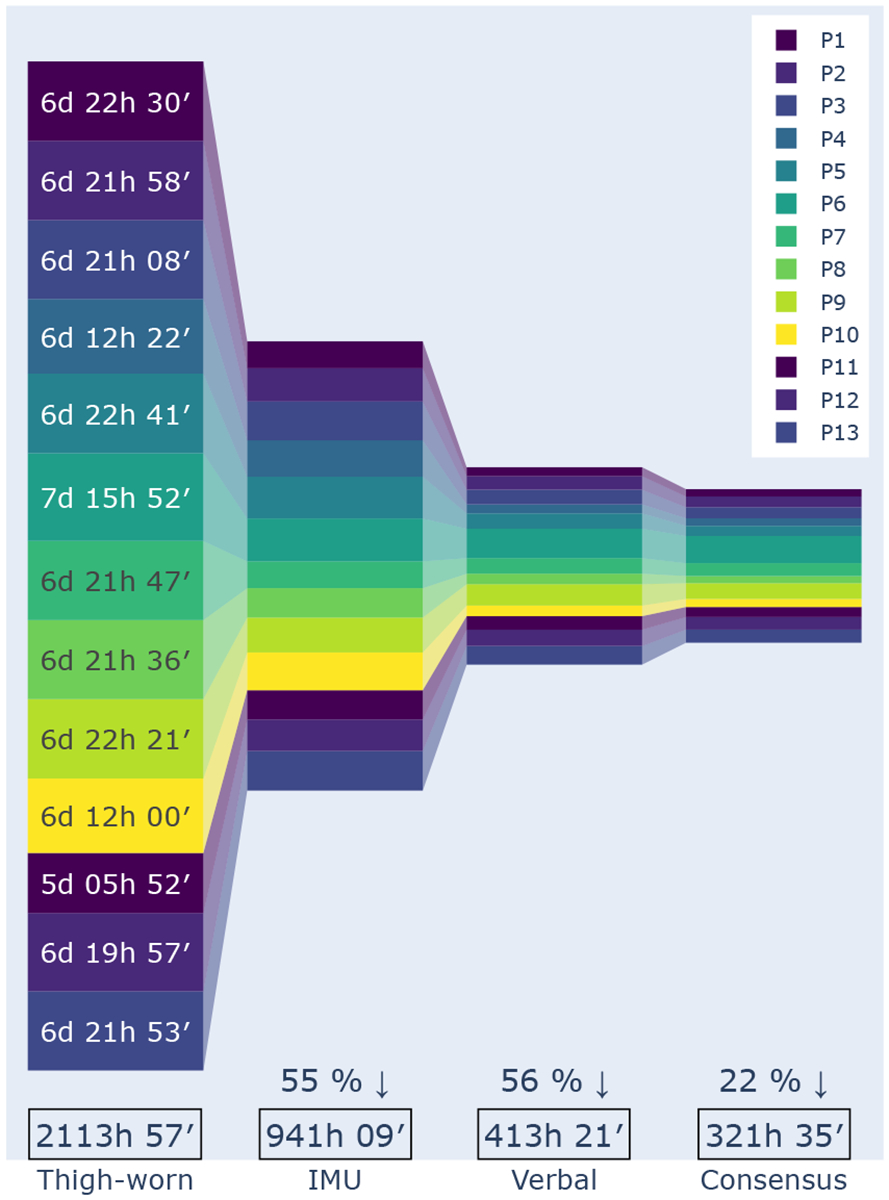
Data shrinkage in consensus labeling. Shown are minutes of raw and labeled data from the thigh-worn sensor, IMU data from the smartwatch, labeled data with verbal reports, and consensus. Epochs where the thigh-worn sensor indicates activity transitions are removed first. Epochs when the smartwatch was unworn are excluded in IMU, Verbal, and Consensus. While this paper focuses on data shrinkage from IMU to consensus-labeled data, thigh-worn sensor data are included to show the time participants contributed data and to support future work on activity labeling with similar wearable sensors.

**Fig. 6.**
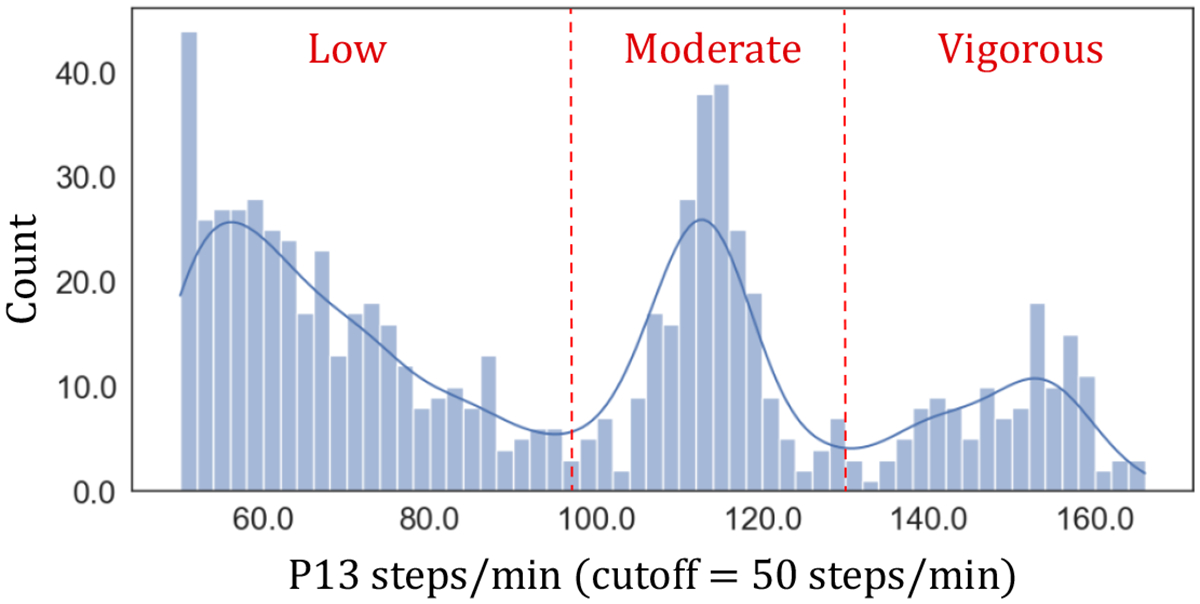
Stepping cadence distribution for P13, showing three peaks and manual thresholds. Bin size = 2; the curved line represents kernel density estimation. Stepping epochs with fewer than 50 steps were excluded for clarity.

We observed that a large portion (21%) of the thigh-worn sensor data included more than one activity label in the span of a minute, the unit of analysis in this paper. These involved either two or three alternating activities. For example, Stepping often alternated with Standing (14%) or with both Sitting and Standing (4%). The labels for these minute-long epochs are based on the activity that is present for more than 30 seconds. Only a small portion (1.3%) of the data included multiple activities, where none of them lasted more than 30 seconds, the threshold used in our analysis. Thus, these data points were excluded. As shown in Figure 5, after this data exclusion we work with a total of 2113 hours and 57 minutes (23 hours and 13 minutes per day per participant on average) of thigh-worn sensor data.

### Excluded verbal reports data.

The largest data exclusion occurs for activity events extracted from voice entries at a 12.8% rate. This includes those that lacked timing information (6.3%), a clear mapping to our hierarchical activity scheme (5.2%), or both (1.3%). Occasionally, participants included activities in their voice entries that occurred during a period where the smartwatch was not worn (19%). Those are also excluded. As shown in Figure 5, after the exclusions, we work with a total of 413 hours and 21 minutes (4 hours and 34 minutes per day per participant on average) of verbal report data.

We triangulated final data from both sources to obtain consensus activity labels in Section 4.2 and explored their potential as ground truth for developing and assessing activity trackers in Section 4.3. These sections rely on findings from our analysis of personalized stepping intensity in Section 4.1, where we utilized cadence distribution to determine thresholds for low, moderate, and vigorous stepping.

### 4.1 Distinguishing between Low, Moderate, and Vigorous Stepping

As noted in Section 3.2.1, it is important to distinguish between various cadence intensities, as older adults can benefit from tracking low-exertion activities along moderate and vigorous activities [93, 94]. We differentiate Low and Moderate from Vigorous Stepping by examining the distribution of stepping cadence for each individual in our study. As shown in Table 3, we provide evidence that cadence thresholds (steps/min) per participant across these three categories are distinct and can benefit from personalization (RQ1). Specifically, we observe that thresholds between Low to Moderate Stepping are in the wide range of 80–105 steps/min for the older adults in our study. All thirteen participants had instances of both Low and Moderate Stepping. Corresponding verbal reports of Low Stepping typically occur within composite activities along Standing and Moderate Stepping. Many include shopping like *“pushing a stroller and picking up items”* (P3) as well as house chores like *“working in the yard”* (P1) and *“walking around the kitchen”* (P10). Fewer refer to sports like *“Completed golf. Good exercise.”* (P1). In contrast, corresponding verbal reports of Moderate Stepping often occur as singleton activities and include terms like *“**moderate** speed”*, *“**moderate** level”*, *“**moderate** effort”*, *“**moderate** pace”*, *“**moderately** fast”*, *“**steady**”*, *“**medium** exertion”*, *“**some** exertion”*, as shown in Table 4. Only two of thirteen participants (P5 and P13) had instances of Vigorous Stepping, a similar rate to reported percentages of people who run and jog in the US [2] with a smaller threshold range of 130–135 steps/min. Indeed, both participants include running in their corresponding verbal reports saying, *“I just finished walking up here and I’m **breathless** because I **ran** a little bit”* (P5) and *“Went for a **run** from 8:10 until 8:40. … it was fairly **intense**”* (P13).

Keywords like *“moderate”* and *“run”* from verbal reports could be leveraged to automatically estimate these personalized boundaries between Low, Moderate, and Vigorous Stepping, *e.g*., by adapting large language models for this task. However, we suspect that this approach alone may not be reliable. For start, such keywords could be missing or be vague *e.g*., we observe that participants often refer to both Low and Moderate stepping, as just *walking*. Even when the keywords are there, they are reported over longer spans of time *e.g*., we observe that Low and Moderate stepping often alternate in a long report where participants include keywords around moderate exertion. Last, perceived levels of exertions found in keywords could misaligned with cadence distribution. While we would expect all self-reported instances of running for the older adults in our study to be closer to vigorous stepping, we find that this is not the case. We observe that other participants (P2, P3, and P9), who also indicate instances of running in their reports, do not exhibit a third cadence peek in their stepping data. For instance, while moderately stepping at a range of 82–92 steps/min, P9 said, *“I’ve been in the pool running and exercising for about 45 minutes.”*

#### 4.1.1 Comparison with MET estimation methods.

**Fig. 7.**
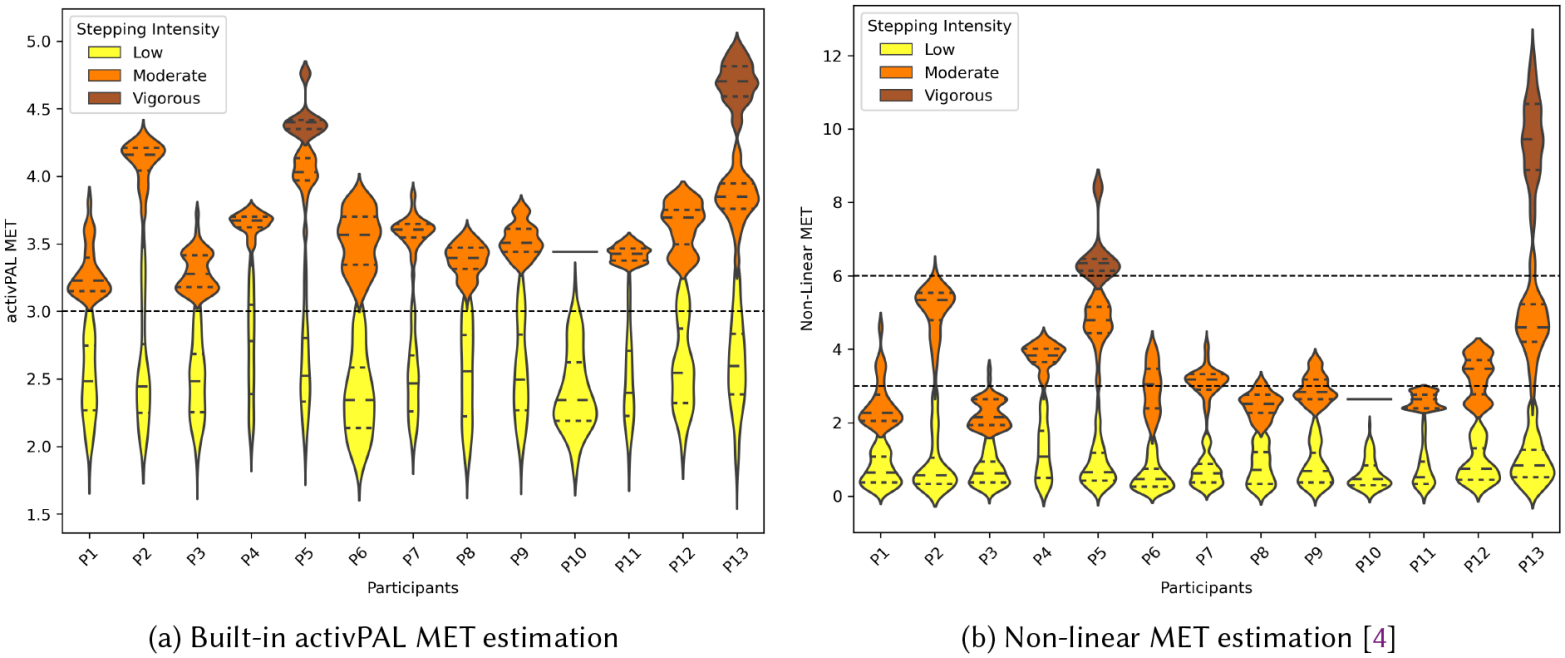
Distribution of estimated METs per participant colored by our personalized stepping intensity method

To compare our method with existing MET estimation approaches, we plot the MET distributions for all participants, colored by our personalized thresholding method (See Figure 7 where Kernel density estimations on each side of the violin plot show the distribution shape). Prior work shows that activPAL MET estimation, based solely on step counts, tends to underestimates METs for vigorous-intensity activities and overestimates them for low-intensity activities [42]. In our dataset, the underestimation is even more pronounced for vigorous stepping. As shown in Figure 7a, low-intensity stepping mostly aligns with the activPAL’s MET estimates of ≤ 2.99, but all vigorous stepping instances are estimated below 6, significantly underestimating the MET for vigorous activity. Due to these biases, we do not use activPAL’s built-in MET estimates for classifying stepping intensities.

More accurate MET estimations have been achieved using linear and non-linear regression with step counts [4] or by incorporating acceleration and heart rate [68]. As shown in Figure 7b, the *MET* = 6 threshold closely aligns with our distribution thresholding method. While this approach better estimates METs for vigorous stepping, such methods often overlook individual variability, particularly among older adults [71]. Sensors that capture elevation, temperature, humidity, as well as heart and respiratory rate, typically found in smartwatches, could be also leveraged when automatically estimating personalized stepping boundaries from cadence. We found that participants’ reports often include perceived effort along with mentions of this information. For instance, they say *“… walking my dog some up hills. Really, really hot.”* (P3) with cadence 0 to 94 steps/min, *“I just had a walk with the dog … It’s warm out and just yucky.”* (P9) with 0 to 68 steps/min, and *“I am still walking on my 5K … breath is a little faster so I’m a little, I’m a little breathless…”* (P5) with 118 to 140 steps/min. We compared our personalized stepping intensity thresholds with the general non-linear MET thresholds from [4] against reported stepping intensities. Our method aligns 18% more closely with verbally reported intensity levels.

### 4.2 Demonstrating the Costs of Consensus Labeling

The costs of obtaining ground-truth data using our approach are multifaceted. Our analysis identifies two main threads of costs: data shrinkage and the effort required from both data contributors and data stewards.

#### 4.2.1 Cost: Data Shrinkage.

Perhaps not surprisingly, we found that a major cost of obtaining ground-truth labels via consensus is significant data shrinkage across participants and activities. Triangulating the two labeling sources yielded an average of only 3 hours and 32 minutes labeled IMU data per day per participant (*σ* = 1 hour and 35 minutes), with just one-third (34%) of the IMU data labeled by consensus overall. This reduction was normally distributed across participants; for instance, more than half (64%) of P6’s IMU data was retained, while only about a fifth (22%) remained for P10. As shown in Table 5, the consensus data covers 78% of verbally reported IMU data, with the highest agreement for P6 (92%) and the lowest for P5 (66%).

**Fig. 8.**
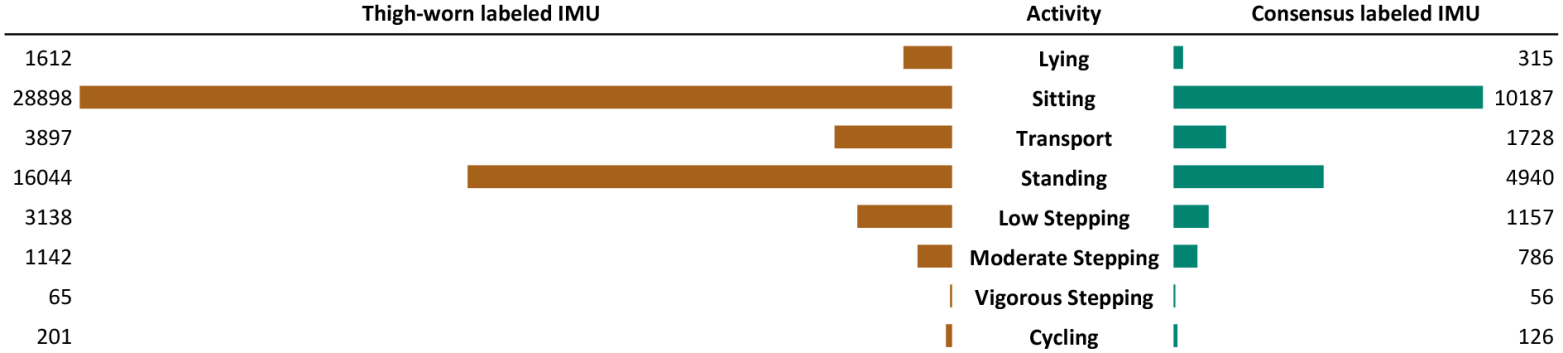
Data shrinkage across activities in consensus. Most Lying data from the thigh-worn sensor occurred during night sleep when participants neither wore the smartwatch nor provided verbal reports. Verbally reported composite activity events primarily (82%) contributed to Standing and Low Stepping. However, about two-thirds of low-intensity activities (Sitting not in transport, Standing, and Low Stepping) were missing due to a lack of verbal reports.

Overall, as shown in Figure 8, the worst shrinkage — leaving us with only 20% of the data — occurred for Lying. Significant shrinkage was also observed for Sitting not in transport, Sitting in transport, Standing, and Low Stepping, leaving 35%, 44%, 31%, and 37% of the data, respectively. In contrast, the shrinkage was less pronounced for high-intensity activities such as Moderate Stepping, Vigorous Stepping, and Cycling, where 69%, 86%, and 63% of the data were retained. This is encouraging, as these activities are relatively rare, allowing us to capture the majority of their instances. We identified two main contributing factors behind this shrinkage and quantified their impact across activities.

##### Lack of verbal reports.

As shown in Table 6, we observe that this cost was not equally distributed across the activities, with the lack of verbal reports being more prevalent in low-intensity activities such as Standing, and Low Stepping with agreement on only 7% and 4% respectively of those instances that the thigh-worn sensor and the smartwatch were worn and participants were reported them explicitly(singleton). This finding is surprising, given prior work showing older adults’ interest in tracking these activities [53, 93] and the associated health benefits [94]. Yet in our study, participants often underreported these activities; even when reported, they are part of composite activities, making it harder to extract labels along the time axis (see Table 6). At the other end of the spectrum, high-intensity activities such as Cycling and Vigorous Stepping had the highest reporting rates — in fact, even exceeding the number of instances detected by the thigh-worn sensor.

##### Lack of agreement between verbal reports and thigh-worn data.

**Fig. 9.**
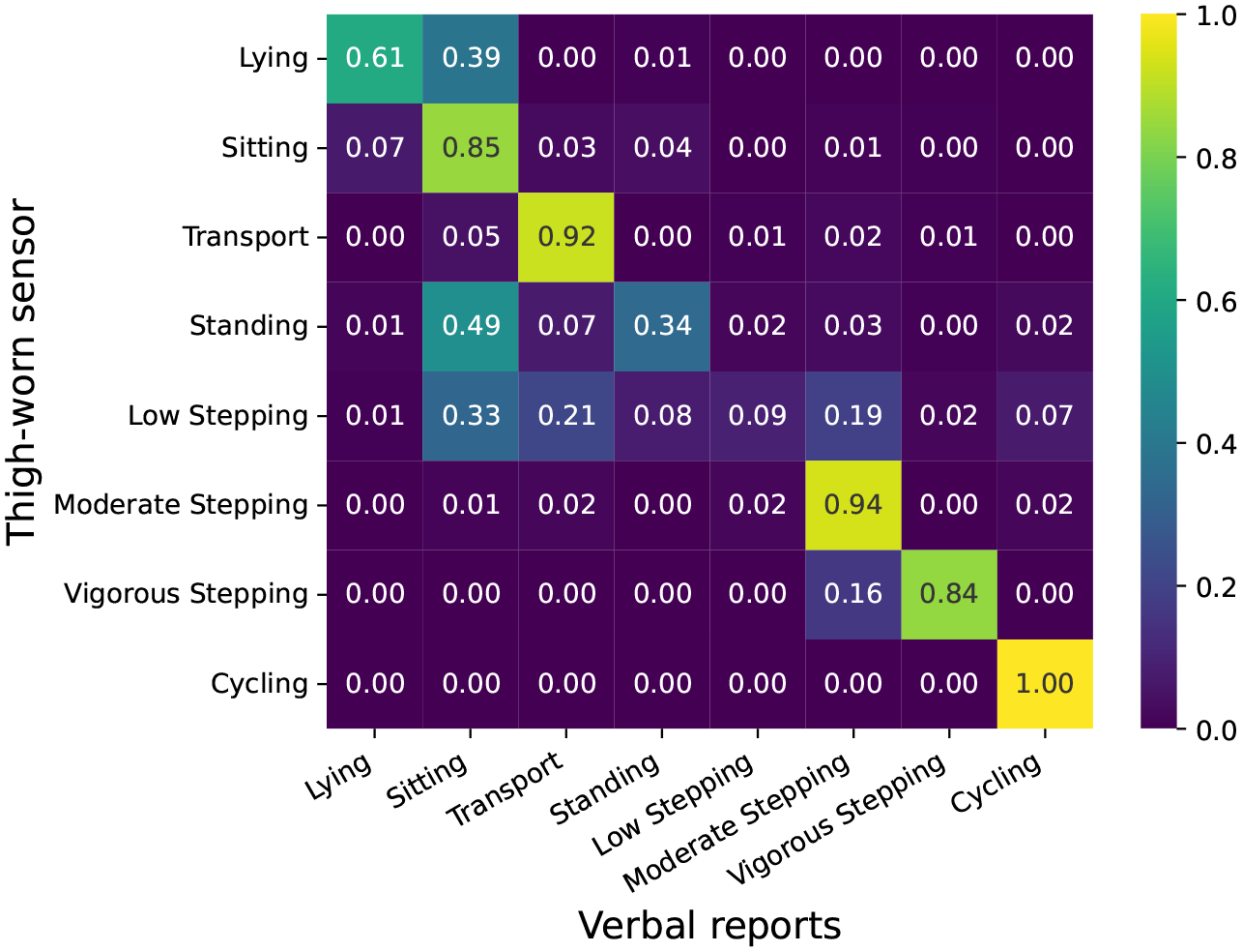
Agreement between verbal reports and thigh-worn sensor classifications for different activity labels. The confusion matrix shows the proportion of verbal report labels aligning with thigh-worn sensor labels, normalized based on the latter.

As shown in the confusion matrix (Figure 9), which includes only singleton verbal reports, Low Stepping, Standing, and Lying show the lowest agreement with the thigh-worn sensor. In contrast, high-intensity activities such as Moderate Stepping, Vigorous Stepping, and Cycling exhibit strong agreement, with alignment rates of 94%, 84%, and 100%, respectively.

Disagreements on instances labeled as Lying by the thigh-worn sensor could be explained by the difference between the actual posture captured by the thigh-worn sensor and the language adopted in the verbal reports that often capture participant intention behind the activity. For example participants sometimes used the term *“lying”* to describe situations where they were in fact sitting. As shown in Figure 9, agreement for Lying is only 61%, with many cases misclassified as Sitting not in transport (39%) due to similar postures. This mismatch is illustrated by P5 and P2, who reported *“lying on the couch…reading and napping”* and *“lying on the floor…watching TV”*, while the sensor recorded Sitting not in transport in both cases. Even identical verbal reports can correspond to different postures: P2, for instance, reported *“reading in bed”* — which was detected as Lying in the morning but Sitting not in transport in the evening.

However, for Standing and Low Stepping, the shrinkage is primarily driven by the lack of agreement between the two sources, with alignment rates of only 34% and 9%, respectively. As shown in the confusion matrix, participants often reported Standing instances as Sitting not in transport, highlighting another common source of mismatch. For instance, a Standing posture was detected by the thigh-worn sensor when P5 and P11 reported *“sitting on a kitchen stool”* and *“sitting on a stool the entire time”*, respectively. We suspect that sitting on a high stool results in a posture that differs from typical sitting, leading the sensor to classify it as standing.

Low Stepping, the activity with the lowest agreement between the two sources, is confused with all other activities. About 20% of them are confused with Moderate Stepping, which could be beyond the effect of thresholding, and more due to participants referring to both Low and Moderate stepping, as just *walking*, discussed in Section 4.1. Another potential explanation could be that Low Stepping is often short in duration and is spread across other activities, many of which are composites. For instance, we observed many cases of Low Stepping during a period that P13 reported as *“Ate dinner from 6:00 o’clock until 9:00 o’clock”* and P1 as *“a 30 minute drive, as sitting …no movement, relaxed”*.

#### 4.2.2 Cost: Effort.

**Fig. 10.**
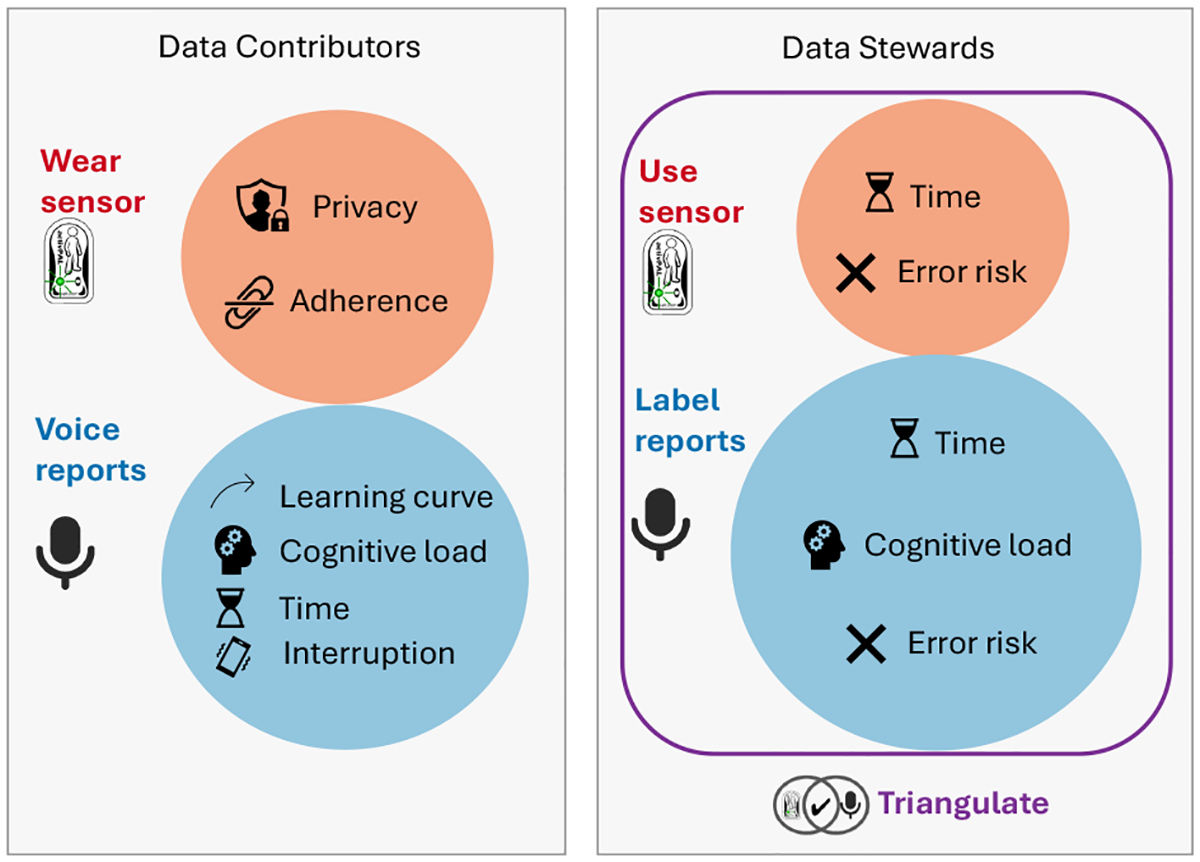
Efforts involved in data collection for data contributors and data stewards.

Our approach facilitates ground-truth data collection in the real world. The implementation of our smartwatch application employs a series of design rationales such as incorporating form factors and interaction modalities that prioritize older adults, streamline the user interface flow for activity reporting, and leverage the flexibility of speech input via natural language. Thus, by design, it aims to impose less burden on data stewards especially when compared to the commonly adopted labeling methods surveyed in Section 2.2. Yet, the procedure remains effort-intensive for data contributors and data stewards. We categorize and, where possible, quantify this cost and surface implications for future human-centered AI approaches for eliciting high-quality data while lowering the burden both on the data contributors and data stewards (see Figure 10).

##### Data contributors.

During a short introductory remote session, participants were instructed to place the waterproof adhesive activPAL patch on their thigh and only remove it 11 days after, at the end of the study. Participants did not have to interact with this sensor, which required no charging for that period. Yet, we observed instances where this happened, albeit a few. Specifically, there were a total of six periods of missing thigh-worn sensor data across three participants, suggesting that the sensor was removed. Only one of the participants (P9) reports removing the sensor, *“I’ve been in the pool running and exercising … I did take off the fitness tracker …”*. When looking at the time window for the other periods, we observe that one of them occurred a few hours before the end of the study and the rest during the night. This indicates that beyond the inherent cost on privacy associated with a 24/7 wear of a sensor, there are additional costs of adherence.

Another heavy lift for participants was the collection of data on the watch. Unavoidably, there was a learning curve for interacting with the device and our smartwatch application. Participants were given a 4-day adaptation period before the 7-day study. Naturally, there is a cognitive load associated with having to remember to charge and wear the watch as well as recall and report activities, their timing, and level of exertion as one goes about their daily living. Each participant spent on average 4 minutes and 15 seconds per day (*σ* = 3 minutes and 15 seconds) just recording verbal reports; we estimate the time spent to be actually a bit longer as participants had to interact with the app and in some cases make multiple attempts at recording not captured by the above numbers. Another way this effort-related cost manifests is in the level of interruptions participants experience, especially when they are prompted to enter a verbal report. We observe that in a day each participant recorded about half of their verbal reports upon being prompted (*μ* = 6.8 reports/day, *σ* = 2.5); their response rate to these prompts was high, about 98.4% of the overall prompts they received that day.

##### Data stewards.

In our study, data stewards’ efforts are also distributed across handling sensor data and verbal reports, with time spent and cognitive load on the first being significantly less than the second in addition to the need for triangulating the two. Analyzing the activPAL thigh-worn sensor data and mapping them to a shared activity scheme and time representation took less than 80 hours. Inherently this process is error-prone as data stewards make decisions and assumptions, albeit somewhat informed. For instance, thresholding was used on the number of seconds for the presence of an activity within a minute (see Section 3.2.1) and on the cadence for distinguishing between Low, Moderate, and Vigorous Stepping (see Section 3.3.1). Manually extracting times and activities from the verbal reports took more than 300 hours. Among the 1357 reported activity events (from a total of 1241 entries), data stewards excluded 82 with incomplete time information, 62 with an unclear mapping to the hierarchical activity scheme, and 17 with both. The insights below from the 1196 labeled and timed activity events, highlight challenges and implications for automating this process *e.g*. with large language models or labeling interfaces informed by older adults reporting styles.

###### Extracting the time span.

We observe 10 time-reporting styles adopted among older adults, shown in Table 7. We observe that a relatively small number (7.3%) of activity events lack time cues (I1-I3), which is promising. Interestingly, these incomplete time cue styles were almost absent for some participants (P6, P9, P10, P11) but roughly equally distributed among the rest.

The majority (7) of the styles include time cues that allow data stewards to calculate and estimate both the start and end times for most (92.7%) activity events. Data stewards integrated manual coding with rule-based programming to extract the time spans. This is promising and motivates further work on automation and in a more guided way to report timing. For instance, the majority of the reports included times cues with respect to a past duration (C1: 32.4%), an update since the last report (C2: 21.9%), or the moment of the recording (C3: 19.1%). This prevalence of C1, C2, and C3 styles was also distributed among the majority of the participants. P11 and P13 were the only exceptions; their reports tended to include time cues within each report with start and end clearly indicated (C4) or start indicated and the report itself recorded at the end of the activity (C6).

###### Assigning activity labels.

**Fig. 11.**
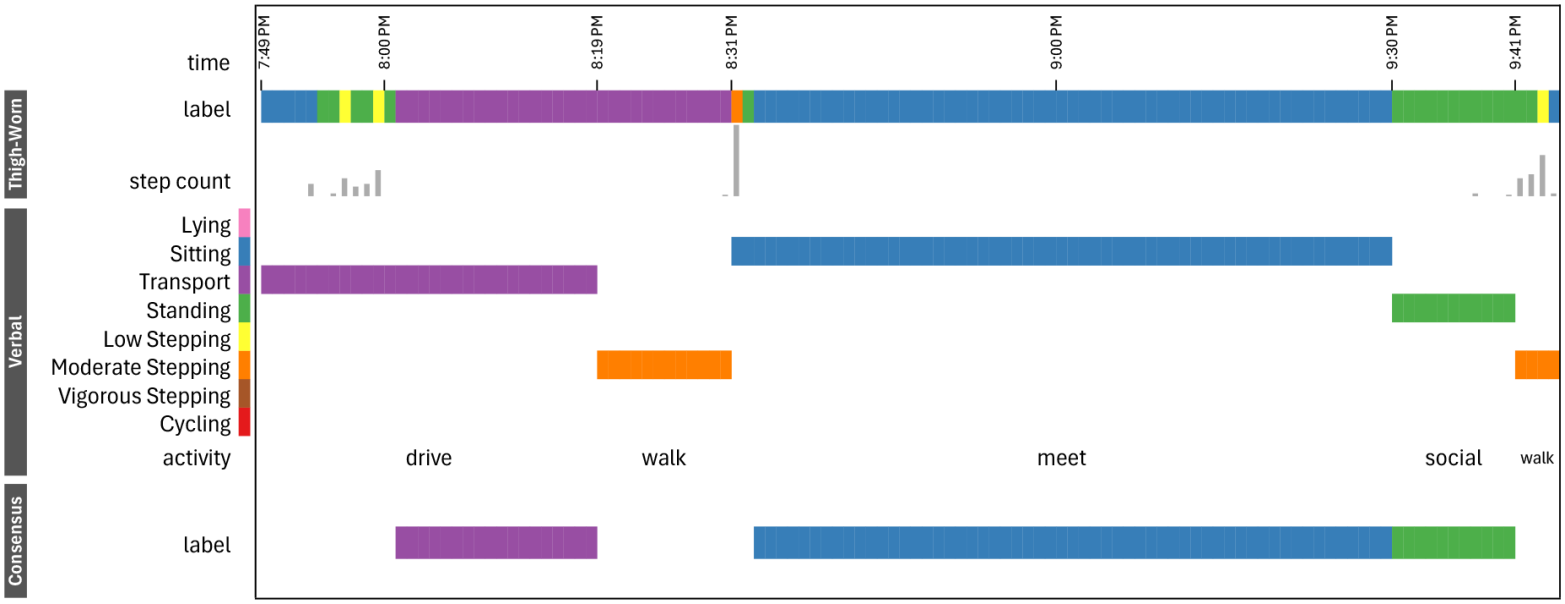
A demonstration of data shrinkage and effort costs in a verbal report from P6. Contrasting the assigned labels from the verbal reports to the thigh-worn sensor labels, indicate that when recalling the activities, P6 merged the Moderate Stepping before and after driving. Despite the efforts of the data steward, this resulted in a consensus where the time span for Sitting in Transport and Standing has shifted and shrunk and the Moderate Stepping has been lost.

Inferring activity labels from the descriptive context in verbal reports required careful attention for data stewards to understand the situation and the activity being performed, akin to a *“detective”* task. One of the challenges is the tendency of older adults in our study (all except P10) to include a series of sequential activities within a single report. For instance, P6 said *“For the past hour and 45 minutes, I have been driving …, walking …, sitting for an hour, chatting … for a few minutes after the meeting. The meeting was from 8:30 to 9:30. And now I’m sitting in my car … Took me 30 minutes to drive. It will take me 30 minutes to drive home. … I chatted after the meeting for about 10 minutes standing … an hour sitting in a meeting, and 15 minutes walking to and from…”*. The challenges in these cases, as illustrated in Figure 11, arise in extracting the time span of these activities which is inherently error-prone as it relies on data contributors’ recall and partial or contradicting information. Overall, there were a few occurrences of this reporting style. Indeed, out of the total 1241 recorded voice entries, only 100 (8%) contained a series of activities where transition times were clearly indicated. Expanding these entries results in 216 individual activity events, bringing the total to 1357 activity events.

The main challenges in assigning activity labels surfaces when data stewards further analyze these activity events with most of them including a single label (singleton: 59%), some including multiple labels (composite: 32%), and few no label at all (undefined: 6%). We observe that this distribution tends to be prevalent across all participants. We already discussed in the data shrinkage cost the challenges related to posture and its associated effect on misalignment between verbal and thigh-worn sensor data. Another reporting style that imposes time and error costs for data stewards is the grouping of activities under a shared purpose. For instance, P13 reported, *“I went grocery shopping …”* which may include driving to the store, shopping, returning home, and arranging groceries. Similarly, *composite* activities like *“gardenning”* and *“playing golf,”* may involve both Standing and Low Stepping. This complexity made it difficult for data stewards to assign labels to these time spans, which account for 436 (32%) of all verbally reported activities. However, in many of these composite cases, data stewards could make an educated guess on possible activity labels and were more generous in their assignment (allowing for the triangulation with the thigh-worn data). The few cases that were deemed as undefined (and thus excluded from the data), involved activities where data stewards had to arbitrarily assign labels. For instance, P6 reported *“Just completed an hour of occupational therapy …”*. In this example, the data stewards could not infer or make an educated guess on the possible activity labels.

### 4.3 Demonstrating Relative Gains of Consensus Labels via Benchmarking an Activity Tracker

We compare thigh-worn sensor, verbal report, and consensus data for benchmarking an activity tracker’s real-world performance. Our analysis reports results on the Google Activity Recognition API that was running on the smartwatches of older adult participants. We identify two main gains with our approach.

#### 4.3.1 Gain: Understanding Label Source Effect.

As shown in Table 8, our analysis indicates that in most cases thigh-worn sensor and verbal report labels result in different AUC scores (indicating true positive rate against the false positive rate at various thresholds) when benchmarking an activity tracker in the real world. This is critical; it allows us for the first time to compare reported performance across publications that opt for one or the other approach. Our results also demonstrate that the performance of the model is higher in consensus data, when the two labeling sources agree, a gain that is more prevalent among more intense activities like Running and Cycling. For instance, we observe that the highest disagreement in Google API performance between the thigh-worn sensor and verbal reports occurs in Running (0.96 vs. 0.72) with consensus data indicating that when the two are in agreement, the Google API performs well though there were very few instances of Running (about 1 hour overall) and Cycling (about 2 hours) in our study.

#### 4.3.2 Gain: Decoupling Labeling Errors from Model Errors.

**Fig. 12.**
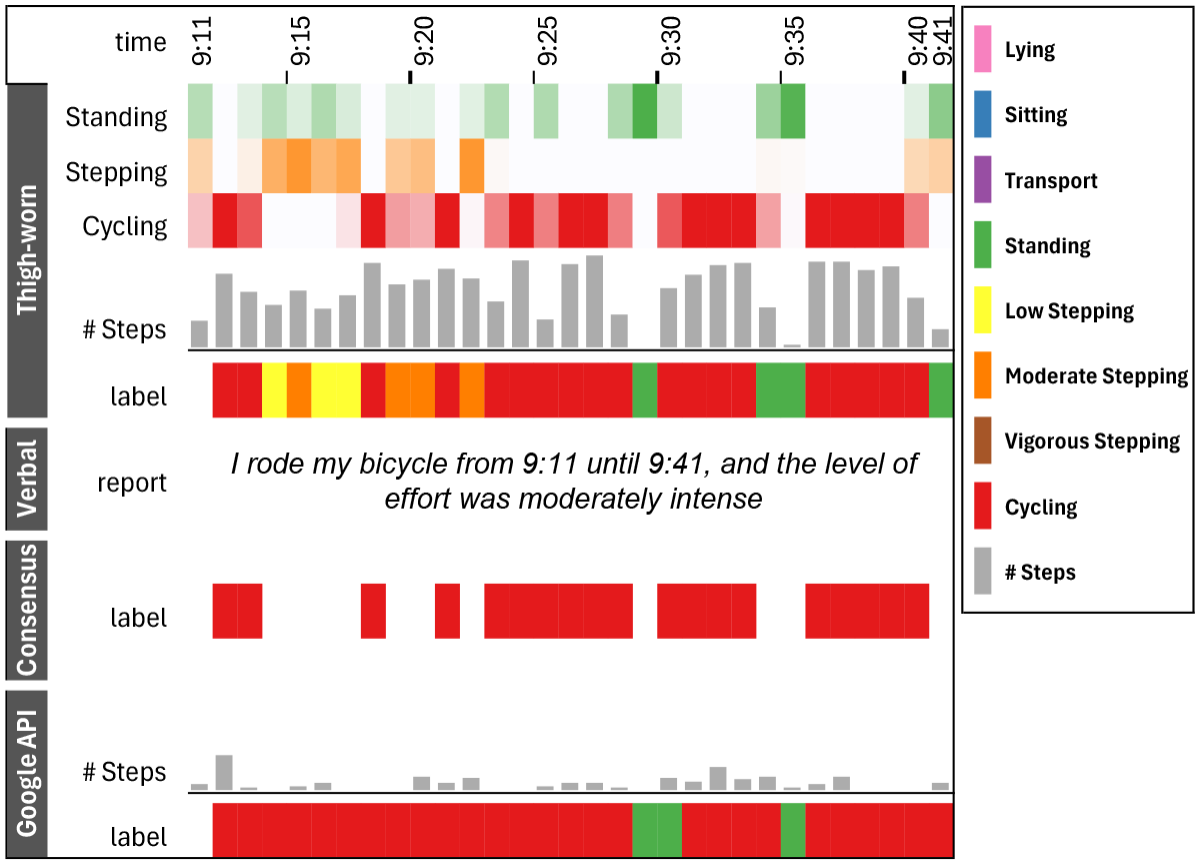
Example of recognition errors due to differences in sensor location when P13 reported *“I rode my bicycle from 9:11 until 9:41, and the level of effort was moderately intense.”*

Having consensus data allows for richer error analysis insights when evaluating an activity tracking model. Specifically, our consensus data helps disambiguate recognition errors contributed by the labels versus the model. In our error analysis, we find that label errors often occur along atypical postures not captured by the thigh-worn sensor. For example, when participants reported *“sitting on a kitchen stool”*, the thigh-worn sensor detected a Standing posture, while verbal reports labeled it as Sitting not in transport; such discrepancies were excluded in the consensus labels. Additionally, label errors often occur along differences in sensor location. The Google API relies on data captured on the wrist in contrast to the thigh-worn sensor. Thus, movement in a specific body joint may better reflect an activity, while other joints remain less active. For instance, during cycling, the thighs are engaged in pedaling, but the wrists, holding the bike’s handlebar, are relatively steady. A similar phenomenon is observed where lower body movements can significantly influence upper body movements, but not vice versa [10]. As shown in Figure 12, the thigh-worn sensor categorized some of the epochs during a 30-minute reported cycling as Standing, Low Stepping, or Moderate Stepping. Although standing may occur during breaks in cycling, such as stopping at an intersection, stepping is less likely in this context. Indeed, this difference in sensor location is also seen in the high #Steps for thing-worn versus low #Steps in Google API, indicating that the thigh-worn sensor in contrast to the wrist-worn sensor often counts pedaling as steps.

When looking at verbal reports, label errors often occur due to misalignment in time cues as precise recalling of the time span of activities can be challenging. We observe that relying solely on the time cues from the verbal reports in an error analysis can be misleading especially for activities that occurred close to each other, as illustrated in Figure 11. Along with computational approaches for automatically detecting transition between activities [8], our consensus labeling can support researchers in disambiguating these cases from other performance errors that can be contributed to the machine learning model.

Specifically, in our evaluation of the Google API with our consensus data, we identify that model errors such as false and missed detection of an activity often occur around being in a vehicle and being still. As shown in Table 9, there are a total of 79 epochs among all participants where the Google API detects that they are Sitting in transport when in fact the consensus indicates that they are Standing. Our manual inspection indicates that 85% of these cases involved the use of a motorized tool, such as motorized kitchen appliances, gardening tools, elevators, or carts. We suspect that these model errors (false positives) are likely explained by the similarities in vibrations between motorized tools and transportation vehicles. Other cases of false detection of Sitting in transport seem to occur a few minutes before or after driving, where our consensus indicates Low Stepping. A more common model error is the missed detection of Sitting in transport, with a total of 802 epochs shown in Table 9. Our manual inspection indicates that these false negatives tend to occur when older adults are sitting in a vehicle that is stopped or idling. Similarly, we look into model prediction around being Still, which Google API defines as “the device is still (not moving)”. Given that the Still label does not provide much information on the older adults’ posture, it is often hard to evaluate the model performance on this prediction with our consensus labels. Specifically, it is hard to tell whether the 3, 1100, and 3187 epochs where Google API predicts as not being Still but the consensus indicates as Lying, Sitting not in transport, and Standing, respectively are false detection (false negatives). Our manual inspection indicates that these cases occur during composite activities such as *“gardening”*, *“carrying/arranging”*, *“preparing food”*, *“cleaning”*, and *“shopping”*, where participants may be moving their upper body. Similarly, it is hard to tell whether the 374 epochs where Google API predicts as being Still but the consensus indicates as Sitting in transport are missed detection (false positives) as the vehicle could sometimes be idle during a ride. But the model errors that we can confidently say that are false positives are the 102, 3, and 2 epochs where Google API predicts as being Still but the consensus indicates as Low Stepping, Moderate Stepping, and Cycling, respectively.

## 5 Discussion

Our exploratory study highlights promising results and future directions for collecting ground-truth human activity data with older adults in real-world settings, aiming to better understand associated challenges. We reflect on key lessons learned, discuss implications for ground-truth data collection methods with older adults and others, and address limitations that may affect the applicability and generalizability of our findings.

### 5.1 Summary of the Findings

#### Personalized cadence intensity thresholds (RQ1).

We investigated the challenges of obtaining ground truth from verbal self-reports and step counts collected by the thigh-worn sensor, focusing on personalized thresholds for low, moderate, and vigorous-intensity stepping. First, we find relying solely on verbal reports to differentiate between low and moderate stepping was problematic, as participants often used the term *“walking”* for both, making clear distinctions rare. Second, cadence thresholds for distinguishing between low and moderate stepping varied widely, from 80 to 105 steps per minute for the older adults in our study. This aligns with prior research on variability in movement patterns for the same activity among older adult individuals [19, 48, 52]. Our findings suggest the potential to automatically estimate these personalized stepping boundaries by combining cadence data with exertion information from verbal reports or sensors that capture additional factors such as elevation, temperature, humidity, and heart and respiratory rates. Although instances of vigorous-intensity stepping (commonly referred to as running) were limited in our study, participants consistently distinguished running in their vocabulary, with this distinction further supported by the cadence thresholds.

#### Costs of obtaining consensus labels (RQ2).

We identify two main costs associated with obtaining ground-truth data using a consensus approach. The first is data shrinkage, with only one-third (34%) of the IMU data labeled through consensus. This issue was observed across all participants and activities, driven by both a lack of labeled data from verbal reports and discrepancies between the thigh-worn sensor data and self-reports. Participants were more likely to report high-intensity activities like cycling and running than low-intensity activities, leading to greater data shrinkage in activities such as lying, sitting, standing, and low stepping. The second cost relates to the efforts required from both data contributors and data stewards. In addition to privacy concerns and time spent reporting, older adults face additional work in wearing the sensor, learning to use the smartwatch, recalling activities, and dealing with interruptions. Meanwhile, data stewards faced substantial cognitive and time demands, spending over 300 hours annotating the verbal reports and using their programming and data analysis skills to process and reach the consensus. Despite this extensive effort—averaging over an hour per consensus-labeled dataset—errors persist due to subjective decisions made when resolving inconsistencies in activity time spans and labels.

#### Relative gains of obtaining consensus labels (RQ3).

We identify two key gains from using consensus labeling. First, we observed a notable improvement in model performance when utilizing consensus data, particularly for high-intensity activities like running and cycling. Additionally, consensus labeling facilitates a deeper understanding of error sources, whether stemming from inaccurate labels or model limitations. For instance, the thigh-worn sensor as ground truth often struggles to accurately identify atypical postures, such as *“sitting on a kitchen stool”*. Differences in sensor placement also lead to discrepancies between model predictions and labels, as seen in cycling and stillness detection. Furthermore, consensus labeling mitigates issues from errors in verbal reports’ activity time cues, which makes relying solely on verbal reports as ground truth problematic. Our analysis also uncovered specific model errors, including instances where the Google API mistakenly interpreted vibrations from motorized tools as indicative of being in a vehicle or failing to detect a stationary vehicle.

### 5.2 Obtaining Ground-Truth Data in Real-World Settings

Our labeling approach captures participants’ interpretations of their activities using our verbal annotation app on smartwatches, while most prior studies rely on data stewards for labeling, either by direct observation or by annotating recorded videos (see Table 1). This method preserves participants’ privacy by avoiding video recordings and better captures their everyday movements in natural settings. We showed that consensus labeling can be used to evaluate activity recognition models and help clarify whether errors originate from the model or the labeling sources. Despite these advancements, challenges remain. Mapping verbal reports to the hierarchical activity scheme is time-consuming and subjective, as annotators may differ in their labeling decisions, time intervals, and overlap resolution potentially differing. Additionally, excluding epochs where sources disagree results in data shrinkage, averaging only 3 hours and 32 minutes per day per participant (see Figure 5).

We observe that participants under-reported low-intensity activities compared to the recorded IMU data (see Table 6), despite older adults’ interest in tracking these activities and the associated benefits [93]. From the data contributors’ perspective, these low-intensity activities, due to their brief and sporadic nature [93], might have been more challenging to delineate and accurately report, especially when interwoven with other distinct activities or when clear start and end times were hard to pinpoint. However, because these activities occur much more frequently than high-intensity activities, the resulting ground-truth data is more balanced, with low-intensity activities still contributing the majority. This could be either a drawback or a benefit depending on the application of the collected ground truth data. For example, balanced data is typically preferred when training machine learning models [23]. However, when evaluating an activity tracker, any data shrinkage represents a loss of valuable information.

Future research directions that may address these challenges include exploring language models to convert verbal reports into activity labels, which could minimize data stewards’ subjectivity [54]. Additionally, developing interfaces that enable users to log their activities using various modalities and devices(*e.g*. drop-down menus, typing, voice entries on smartphones and smartwatches) or using advanced signal processing techniques to identify the activity transition times [8] and provide model initial predictions to participants for review [33], could help alleviate the burden of self-reporting. The latter approach may reduce the overall number of labels needed while capturing essential labels that directly enhance model performance.

### 5.3 Developing Teachable Activity Trackers

We explored the challenges of obtaining ground-truth activity data with older adults, aiming to pave the way for future work. By leveraging the design insights from this study and incorporating machine learning techniques, the approach could be expanded into a teachable activity tracker. We identified two main threads in developing a teachable activity tracker: personalization and data collection.

#### 5.3.1 Personalization.

Within personalization, we encountered three significant challenges. (1) Our study revealed significant variations in user interpretations of activities and exertion levels, as evidenced by the disparities in stepping intensities and distinctions between lying and sitting. We addressed this through two approaches: a verbal reporting app on a smartwatch and individual cadence thresholds. User perception of exertion levels adds complexity, being influenced by multiple factors such as activity intensity, environment, and previous workload. While subjective reporting captures these nuances, objective measurements like heart rate could provide standardized data. Future research should explore integrating both approaches. (2) Another challenge lies in accommodating user preferences for granularity, for instance, whether they prefer to see a grocery shopping event as a whole or as multiple short instances of driving, walking, standing, and bending. This preference may differ between users and even for the same user over time. Potential solutions include considering multiple granularity levels in data labeling and model training or initially focusing on the highest granularity and subsequently inferring longer activity periods based on finer detected activities. (3) Another personalization factor that is important for older adults is to track low-exertion activities as they contribute to physical health. However, current technologies struggle to capture these sporadic, short activities effectively [93]. In many low-exertion activities, such as *“washing dishes”*, one part of the body may be moving while other parts are relatively inactive. In such cases, the smartwatch might detect movement, while the thigh-worn sensor might classify it as standing. Triangulating with verbal reports can help identify a new separate set of data points that could be used to train a personalized activity tracker that better classifies these composite activities for older adults.

#### 5.3.2 Data Collection and Labeling.

Another challenge in developing a teachable activity tracker lies in minimizing the burden of data collection while ensuring accurate recordings of natural behaviors. However, sustainably collecting sufficient data to train an activity recognition model remains a challenge. Our 7-day study yielded only 3 hours and 32 minutes of ground-truth data per day per participant on average, highlighting the need for more efficient data collection methods. One notable obstacle is the inconsistency and variety in reporting time cues, with our study revealing 6.3% of verbally reported activity events of incomplete timing and seven different styles of time reporting. Moreover, 19.5% of reports (C3 category) only provided information for the moment of recording, which is not helpful in understanding the entire interval of an activity event. To address these timing challenges, future research could explore several approaches: (1) guiding participants to follow specific time reporting styles, (2) identifying activity start/end times through other means such as additional sensors like activPAL, Google API, or a trained model [8], and only asking users about the type of activity during that period, or (3) prompting participants to complete time information later in the day.

To overcome the two threads of challenges: personalization and data collection, future research could investigate several approaches. By applying federated learning [35, 61, 96] the user data will remain in the user device while collaborating in training a global model and build up a personalized model locally, as well as data augmentation [60, 91, 99], self-supervised [70, 84, 97], and semi-supervised [69, 100] methods to leverage unlabeled data effectively with few or no labeled data. These avenues of research could lead to the development of personalized privacy-preserving activity tracker for older adults in real-world settings that requires less labeling effort.

### 5.4 Complexity, Subjectivity, and Ground Truth

We face several challenges in assigning real-world activities to a set of limited labels. For instance, composite activities like *gardening* and *shopping*, which are often low effort activities particularly interesting to track for older adults, with multiple labels assigned (often standing and low stepping), cover 32% of our verbally labeled data. Moreover, we observed the complexity of labeling activities with atypical postures that fall between our categories, such as *“sitting on a kitchen stool”* where the thighs are not parallel to the surface, and the posture is somewhere between sitting and standing. These cases raise questions about what constitutes the “true” label and whether a data contributor’s perception should override the thigh-worn sensor output. This example also highlights the limitations of the consensus labeling approach, as such disagreements lead to data shrinkage. Another example of composite activities like *“emptying the dishwasher”*, which involves bending, presented similar challenges. The challenge of mapping real-world data to a set of limited labels aligns with recently observed challenges in the data quality literature, where the existence of a binary truth is questioned [12]. The process of mapping real-world postures to a limited set of labels is inherently subjective. To minimize this subjectivity and towards consistency, we implemented a coding scheme agreed upon by three researchers. Dedicated labels for the mentioned composite activities like *gardening* and *shopping* may help solve the problem. Yet, distinguishing between them seems difficult for activity recognition models, as both involve multiple instances of standing, stepping, and bending. Future research could focus on these complex activities by training models over extended periods to recognize the patterns in transitions between postures, potentially improving the recognition and differentiation of these compound activities.

### 5.5 Limitations

Our study is exploratory and subjective in nature, and as such, the findings should not be considered definitive. Rather, the research provides a rich set of observations and insights that generate hypotheses requiring further investigation. While our results offer valuable contributions, several limitations must be acknowledged.

#### Small and Homogeneous Participant Group.

One major limitation is the small size of the study group, consisting of only 13 participants. Furthermore, our participants had relatively homogeneous characteristics: they had no disabilities or motor impairments, were all English speakers, had at least a bachelor’s degree, and were motivated to participate in the study. This homogeneity limits our ability to generalize the findings to a broader population, especially when considering individuals with disabilities, different levels of education, or varying cultural and linguistic backgrounds. Future research should include larger and more diverse samples, incorporating participants with different socioeconomic statuses, linguistic backgrounds, and physical abilities to validate and expand upon our findings.

#### Dependence on Verbal Input for Activity Reporting.

Another limitation stems from the reliance on verbal reports on our smartwatch app as the sole input modality for activity reporting. We employed verbal reports to give participants maximum flexibility to describe their activities in their own words. Using alternative methods, such as drop-down menus, typing, and video logging on various devices like mobile phones, smart speakers, or laptops, may have significantly altered our results. By expanding input methods in future studies, a more comprehensive picture of activity reporting could emerge, addressing some of the errors introduced by verbal self-reports alone.

#### Limitations of the Cadence Thresholds Method.

It is also worth noting that the effectiveness of the cadence thresholds method in distinguishing stepping intensities relies on having sufficient data for all three stepping subcategories for each participant. In cases where participants did not exhibit the full range of stepping intensities, the method may not yield reliable thresholds, further impacting the accuracy of activity recognition in those instances.

#### Technological Advancements and Evolving User Expectations.

Finally, with the rapid advancements in activity tracking technology, user experience and expectations are continually evolving. While our findings provide valuable insights into current challenges and future directions, their validity may change as users become more familiar with newer technologies. As a result, future research will need to revisit these findings to account for technological progress and its impact on user interaction and data collection.

## 6 Conclusion

This study explored ground-truth activity data collection with older adults through verbal reports, a thigh-worn sensor, and a consensus labeling approach in real-world settings. We reviewed existing labeling methods and proposed a low-burden, privacy-conscious data collection strategy. Our work contributes empirical evidence on labeling approaches, offering a comparative review and insights into consensus labeling as a proxy for ground truth. While consensus labeling mitigates some data collection burden, it still demands cognitive and time investment from both contributors and data stewards. Despite the data shrinkage introduced by consensus labeling, the resulting data quality is advantageous for model evaluation, as consensus labels reduce noise and help isolate errors stemming from either labeling discrepancies or model limitations. Additionally, we emphasize the need for personalized tracking solutions by demonstrating substantial variation in cadence thresholds for stepping intensities among older adults. Moving forward, we aim to leverage our findings to develop teachable activity trackers tailored to older adults, enhancing the accuracy of capturing individual activity patterns. Ultimately, refining activity tracking methods can foster a more active and engaged lifestyle for older adults, contributing to healthier aging outcomes.

## Figures and Tables

**Table 1.** Characteristics of related studies that collect ground truth and sensory data for activity recognition of older adults. We use different symbols to represent data contributors (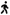) and data stewards (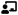) and the following abbreviations for the sensors: Accelerometer (Acc), Gyroscope (Gyro), Magnetometer (Mag), Temperature (Temp), Air Pressure (Pr), Heart rate (HR), Passive Infrared (PIR), Metabolic equivalent (MET), Electrodermal activity (EDA), Photoplethysmography (PPG), Electromyography (EMG), Electrocardiogram (ECG).

		[24]	[79]	[34]	[20]	[72]	[45]	[92]	[10]	[46]	[80]	[78]	[66]	[36]	[13]	[49]	[88]	this
Data Contributors	# People	12	14	8	17	45	5	21	22	15	20	25	253	79	247	20	18	13
	Age Mean	71.8	74.6	76.5	74	50.8	74	-	85.5	66.4	-	-	61.7	75	72.4	75.6	79.6	71.1
	Age Min	65	66	70	-	-	69	60	77	57	24	-	20	65	60	56	70	61
	Age Max	78	86	83	-	-	82	74	93	77	86	-	89	90	89	90	95	90
	Sex (F)	-	10	-	5	23	2	11	19	7	-	-	158	69	142	7	9	10
	Sex (M)	-	4	-	12	22	3	10	3	8	-	-	36	10	46	13	9	3
Activities	# Activities	11	4	5	6	6	1	17	13	6	-	14	33	1	33	2	6	8
	Lying	•	•	•	•	•		•	•	•	•						•	•
	Sitting		•	•	•	•		•	•	•	•	•					•	•
	Transport																	•
	Standing		•	•	•	•		•	•	•	•	•					•	•
	Stepping	•	•	•	•	•		•	•	•	•	•	•		•	•	•	•
	Cycling																	•
	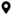 Lab/Clinic		•		-	•					•		•		•	•		
	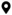 Home	•		•	-	•	•	•	•	•		•					•	
	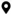 In-the-Wild													•				•
	Goal: (I)ADL	•	•	•		•		•	•	•	•	•	•	•	•	•	•	•
	Goal: Fall						•	•										
	Goal: Fitness														•			•
	Goal: Lifestyle				•	•											•	•
	Goal: Other		•			•								•		•		
Sensor Types	Wearable: Acc	•	•	•	•	•	•	•	•	•	•	•	•	•	•	•	•	•
	Wearable: Gyro				•	•	•	•		•	•			•				•
	Wearable: Mag							•			•							•
	Wearable: Pr	•				•		•		•								
	Wearable: Temp	•						•	•			•		•				
	Wearable: HR										•	•		•				•
	Wearable: MET												•		•			
	Wearable: EDA								•			•		•				
	Wearable: PPG								•									
	Wearable: EMG										•							
	Wearable: ECG										•							
	Ambient: PIR							•	•			•						
	Ambient: Object								•		•							
	Ambient: Laser									•								
Wearable Sens. Loc.	Worn on Wrist	•						•	•	•	•	•	•	•	•			•
	Worn on Thigh			•							•						•	•
	Worn on Head												•		•			
	Worn on Chest		•	•			•				•							
	Worn on Waist			•	•	•										•	•	
	Worn on Ankle										•							
	Worn on Arm										•							
Labeling & Ground truth	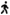 Pick Activity					•												
	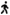 Note Down						•											
	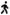 Voice Report																	•
	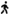 Wear Sensor																	•
	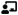 Label in Sync	•	•	•				•			•	•	•	•	•			
	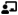 Label Videos				•				•	•	•	•				•	•	
	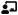 Label Voices																	•
	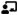 Use Sensors									•								•
	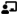 Triangulate									•								•

**Table 2.** Participants’ demographic information.

Partic.	Age (Gen.)	Education	Tech proficiency
P1	61 (M)	Bachelor’s	Very confident
P2	67 (W)	Bachelor’s	Enjoy the challenge
P3	77 (W)	Ph.D./M.D.	Very confident
P4	70 (M)	Bachelor’s	Enjoy the challenge
P5	81 (W)	Master’s	A little apprehensive
P6	79 (W)	Master’s	Very confident
P7	69 (W)	Master’s	Enjoy the challenge
P8	90 (W)	Master-level	Enjoy the challenge
P9	62 (W)	Master-level	Very confident
P10	62 (W)	Bachelor’s	Very confident
P11	67 (W)	Master-level	Enjoy the challenge
P12	75 (W)	Master’s	Very apprehensive
P13	64 (M)	Master’s	Enjoy the challenge

**Table 3.** Stepping thresholds (steps/min) based on visual inspection of the stepping cadence distribution.

	P1	P2	P3	P4	P5	P6	P7	P8	P9	P10	P11	P12	P13
moderate (steps/min)	80	100	80	90	100	80	80	80	90	90	90	85	90
vigorous (steps/min)	-	-	-	-	135	-	-	-	-	-	-	-	130

**Table 4.** Moderate Stepping examples in verbal reports with corresponding stepping cadence ranges for the given period.

ID	steps/min	verbal report
P2	[112,134]	I took a **walk** from 7:00 to 7:30. **Moderate** speed.
P2	[124,134]	I took a **brisk walk** around the block
P3	[56,94]	I’m just back home after **walking my dog** for 30 minutes …**some exertion**.
P4	[86,110]	Just **walked** two and a half miles at three miles an hour. **Moderate** effort
P5	[114,124]	**walking** at a **moderately fast** pace, but not enough to be heavily exerting.
P6	[66,114]	Just return from 35 minute **walk**. **Moderate** level of effort.
P7	[78,102]	I just returned from a 30 minute **walk, fairly easy** paced, **moderate** effort …
P8	[80,96]	I’m not going real fast, but. **Steady**. I’m going up a hill.
P9	[76,108]	I had about an hour of **medium exertion**, **walking** around.
P10	[78,102]	I’ll be **walk**, going into an office and sitting down for a dental appointment.
P13	[96,116]	I **walked** from 11:20 till 12:00 o’clock at a **moderate pace**.

**Table 5.** Data shrinks as we find the consensus labels. The number of collected reports and their durations at each stage.

ID	thigh-worn	IMU	verbal reports			consensus	agreement between verbal & thigh-worn
	span (min)	span (min)	span (min)	span/activity average (min)	count	span (min)	
P1	9990	3381	1113	16	69	915	82%
P2	9958	4183	1707	20	86	1351	79%
P3	9908	4869	1843	14	133	1384	75%
P4	9382	4581	1128	16	71	957	85%
P5	10001	5253	1921	17	112	1270	66%
P6	11032	5382	3700	14	261	3420	92%
P7	9947	3377	1960	27	72	1619	83%
P8	9936	3692	1317	14	96	895	68%
P9	9981	4391	2690	53	51	1972	73%
P10	9360	4754	1332	15	89	1022	77%
P11	7552	3675	1701	45	38	1260	74%
P12	9837	3955	2050	30	69	1580	77%
P13	9953	4976	2339	25	94	1650	71%
**Total**	**126837**	**56469**	**24801**	**20**	**1241**	**19295**	**78%**

**Table 6.** The data volume shrinks as consensus is reached. Most Lying data, captured by the thigh-worn sensor, occurred during night sleep when participants neither wore the smartwatch nor verbally reported their activity. Verbally reported activity events annotated as ‘composite’ mostly contribute to Standing and Low Stepping. However, about two-thirds of low-intensity activities such as Sitting not in transport, Standing, and Low Stepping seem not as important to track for older adults. Composite activity reports only contribute to the consensus when they match sensor data, mostly for Standing and Low Stepping.

Activity	thigh-worn	thigh-worn labeled IMU	verbal-reports labeled IMU	consensus	consensus/thigh-worn labeled IMU
Lying	43940	1612	918	315	20%
Sitting	47230	28898	9935	10187	35%
Transport	4535	3897	2058	1728	44%
Standing	24981	16044	1190	4940	31%
Low Stepping	4493	3138	122	1157	37%
Moderate Stepping	1360	1142	759	786	69%
Vigorous Stepping	65	65	99	56	86%
Cycling	233	201	264	126	63%
Composite & Undefined			9456		
**Total**	**126837**	**54997**	**24801**	**19295**	**35%**

**Table 7.** Time-reporting styles in participants’ verbal reports, where data stewards attempt to extract the interval of an activity event by combining available information on start, end, and duration.

	Type Description		Count (%)	Example
Complete time cues	C1	**End** of the activity is the time of the report **Duration** of the activity is mentioned in the report **Start** of the activity is estimated (end - duration)	440 (32.4%)	*“For the* ***past 20 minutes*** *I’ve been driving to an appointment and the intensity level was low.”* P9
	C2	**Start** of the activity is the time of the previous report **End** of the activity is the time of the report	297 (21.9%)	***“Since my last report****, I’ve been sitting on the sofa and just relaxing, watching television.”* P7
	C3	The activity is being done **at the moment** of the report. No information about start, end, or duration is given.	259 (19.1%)	*“I’m just kind of hanging out, watching the news* ***right now****.”* P8
	C4	**Start** of the activity is mentioned in the verbal report **End** of the activity is mentioned in the verbal report	87 (6.4%)	*“****From 5:00 to 7:00 PM*** *was doing yard work. Moderate exercise. Nothing heavy or strenuous.”* P1
	C5	**Start** of the activity is estimated based on information often spread across multiple verbal reports **End** of the activity is estimated based on information often spread across multiple verbal reports	69 (5.1%)	*“Rode about 5.6 miles over roughly 36 minutes, ending maybe 15, 20 minutes ago.”* P4
	C6	**Start** of the activity is mentioned in the verbal report **End** of the activity is the time of the report	59 (4.3%)	*“So* ***since about 5:00 o’clock****, I’ve been at happy hour/dinner, and, and I have been sitting most of the time. Just enjoy my family’s company.”* P11
	C7	**Start** of the activity is the time of the report **Duration** of the activity is mentioned in the report **End** of the activity is estimated (start + duration)	47 (3.5%)	*“I’m starting out on a casual walk for the* ***next 20 minutes*** *or so. I will report it.”* P5
Incomplete or no time cues	I1	**No information** about start, end, or duration is given	52 (3.8%)	*“After biking home from the co-op and all day I have put away all of the supplies and the bulk items and so forth, that I purchased.”* P4
	I2	**Start** of the activity is the time of the report No information about **end** and **duration** is given.	33 (2.4%)	*“I just sat down* ***to*** *read the newspaper.”* P13
	I3	**End** of the activity is the time of the report No information about **start** and **duration** of the activity is given.	14 (1%)	*“I’ve* ***just finished*** *carrying two gallon jugs into the house from outside so that I can water the indoor plants.”* P5

**Table 8.** AUC per label of the Google API labels evaluated based on the thigh-worn sensor, verbal reports, and consensus labeling sources. Considering the consensus labels as the ground truth, the Google API reliably detects Running and Cycling. However, it falls slightly short of detecting Still.

Google API labels	thigh-worn	verbal	consensus
STILL	0.77	0.80	0.80
(sedentary: lying)	–	–	–
(sedentary: sitting: not transport)	–	–	–
(upright: standing)	–	–	–
IN_VEHICLE (sedentary: sitting: transport)	0.86	0.83	0.89
ON_FOOT (upright: stepping)	0.83	0.86	0.87
WALKING (upright: stepping: low & moderate)	0.82	0.85	0.86
RUNNING (upright: stepping: vigorous)	0.96	0.72	1.00
ON_BICYCLE (cycling)	0.98	0.91	0.99

**Table 9.** False positives and false negatives, indicated in bold, in Google API regarding being in a vehicle and being still.

		Google API			
		IN_VEHICLE	IN_VEHICLE	STILL	STILL
**Consensus label**	sedentary: lying	21		3	18
	sedentary: sitting: not transport	4574	**41**	*1100*	3515
	sedentary: sitting: transport	**802**	1034	1462	*374*
	upright: standing	4579	**79**	*3187*	1471
	upright: stepping: low	1114	**39**	1051	**102**
	upright: stepping: moderate	759		756	**3**
	upright: stepping: vigorous	56		56	
	cycling	132		130	**2**

## Box

CCS Concepts: • **Human-centered computing** → **Empirical studies in ubiquitous and mobile computing**; *Empirical studies in HCI*; *Ubiquitous and mobile computing design and evaluation methods*.
